# Supplementary material for: A Media Analysis of the COVID-19 Tobacco Sales Ban in South Africa
Source: Int J Environ Res Public Health. 2023 Sep 7;20(18):6733. doi: 10.3390/ijerph20186733 (PMC10531267; doi:10.3390/ijerph20186733)
Supplement: Supplementary file 1 [file ijerph-20-06733-s001.zip › Table S1.pdf]

Article No.	Date of publication	Outlet name	Author(s)	Title of article	URL
1	2020-08-10	BizNews	Jarryd Neves	Tobacco ban: Let's call an end to this failed experiment	https://www.biznews.com/inside-covid-19/2020/08/10/tobacco-ban
2	2020-08-04	BizNews	Staff Reporter	Pick n Pay warns 50% drop in earnings; alcohol, tobacco to blame	https://www.biznews.com/sa-investing/2020/08/04/pick-n-pay
3	2020-06-28	BizNews	Staff Reporter	NDZ wins battle for tobacco sales ban – Pretoria High Court	https://www.biznews.com/briefs/2020/06/28/ndz-wins-battle-for-tobacco-sales-ban-pretoria-high-court
4	2020-08-14	BizNews	Jackie Cameron	How tobacco bosses play politicians: See this 8-point plan	https://www.biznews.com/undictated/2020/08/14/tobacco-bosses-corruption
5	2020-10-28	BizNews	Staff Reporter	Revealed: At least R300bn in tax revenue disappears through job losses, tobacco ban #MTBPS	https://www.biznews.com/budget/budget-2020/2020/10/28/tax-revenue
6	2020-05-28	BizNews	Alec Hogg	Hearing the real reason for SA Government's legal tobacco ban	https://www.biznews.com/undictated/2020/05/28/reason-government-legal-tobacco-ban
7	2020-05-26	BizNews	Alec Hogg	Inside Covid-19: R249pm medical aid for 1.2m household workers; Black tobacco farmers say Govt supporting crooks. Ep 39	https://www.biznews.com/inside-covid-19/2020/05/26/covid-19-medical-aid-tobacco
8	2020-08-24	Business Tech	Staff Reporter	Dlamini-Zuma can't promise that the cigarette ban won't return: report	https://businesstech.co.za/news/government/427926/dlamini-zuma-cant-promise-that-the-cigarette-ban-wont-return-report/
9	2020-05-31	Business Tech	Staff Reporter	Government wants 100% smoking ban in public areas	https://businesstech.co.za/news/trending/403497/government-wants-100-smoking-ban-in-public-areas/
10	2020-08-06	Business Tech	Staff Reporter	South Africa's ban on alcohol and cigarettes has not yet served its purpose: Mkhize	https://businesstech.co.za/news/business/423558/south-africas-ban-on-alcohol-and-cigarettes-has-not-yet-served-its-purpose-mkhize/

11	2020-05-07	Business Tech	Staff Reporter	The World Health Organisation on the coronavirus peak, ending lockdown, and the smoking ban in South Africa	https://businesstech.co.za/news/government/395989/the-world-health-organisation-on-the-coronavirus-peak-ending-lockdown-and-the-smoking-ban-in-south-africa/
12	2020-07-01	Business Tech	Staff Writter	Ramaphosa on South Africa's most controversial issues – including the sale of cigarettes, and the death penalty	https://businesstech.co.za/news/government/412681/ramaphosa-on-south-africas-most-controversial-issues-including-the-sale-of-cigarettes-and-the-death-penalty/
13	2020-06-18	Business Tech	Staff Reporter	Alcohol and tobacco sales are 'still open for debate': Ramaphosa	https://businesstech.co.za/news/government/408875/alcohol-and-tobacco-sales-are-still-open-for-debate-ramaphosa/
14	2020-08-07	Business Tech	Staff Reporter	5 important things happening in South Africa today	https://businesstech.co.za/news/government/423644/5-important-things-happening-in-south-africa-today-856/
15	2020-08-26	Business Tech	Staff Reporter	Dlamini-Zuma and Fita settle court battle over South Africa's cigarette ban – here are the conditions	https://businesstech.co.za/news/lifestyle/428856/dlamini-zuma-and-fita-settle-court-battle-over-south-africas-cigarette-ban-here-are-the-conditions/
16	2020-07-17	Business Tech	Staff Reporter	How much money government is losing in tax each month because of South Africa's alcohol and tobacco ban	https://businesstech.co.za/news/finance/416987/how-much-money-government-is-losing-in-tax-each-month-because-of-south-africas-alcohol-and-tobacco-ban/
17	2020-07-21	Business Tech	Staff Reporter	South Africa's cigarette ban could backfire spectacularly: research	https://businesstech.co.za/news/lifestyle/418429/south-africas-cigarette-ban-could-backfire-spectacularly-research/
18	2020-08-14	Business Tech	Staff Reporter	Counting the cost of South Africa's alcohol and cigarette sales ban	https://businesstech.co.za/news/business/425334/counting-the-cost-of-south-africas-alcohol-and-cigarette-sales-ban/

19	2020-05-06	Business Tech	Staff Reporter	Tobacco giant drops plan to sue government over South Africa’s cigarette ban	https://businesstech.co.za/news/business/395621/tobacco-giant-drops-plan-to-sue-government-over-south-africas-cigarette-ban/
20	2020-08-04	Business Tech	Staff Reporter	Pick n Pay speaks out against ‘confusing, contradictory’ alcohol and cigarette ban	https://businesstech.co.za/news/business/422438/pick-n-pay-speaks-out-against-confusing-contradictory-alcohol-and-cigarette-ban/
21	2020-06-08	Business Tech	Staff Reporter	Here’s what will decide what ends South Africa’s cigarette sales ban: Ramaphosa	https://businesstech.co.za/news/business/405733/heres-what-will-decide-what-ends-south-africas-cigarette-sales-ban-ramaphosa/
22	2020-07-24	Business Tech	Staff Reporter	Another blow for business in court battle against South Africa’s cigarette sales ban	https://businesstech.co.za/news/lifestyle/419573/another-blow-for-business-in-court-battle-against-south-africas-cigarette-sales-ban/
23	2020-08-07	Business Tech	Staff Reporter	Government imposed the smoking ban to free up 16 ICU beds: Tobacco company	https://businesstech.co.za/news/government/423862/government-imposed-a-smoking-ban-to-free-up-16-icu-beds-tobacco-company/
24	2020-05-12	Business Tech	Staff Reporter	Alcohol and tobacco ban hurting Pick n Pay as it counts the cost of Covid-19	https://businesstech.co.za/news/business/397015/alcohol-and-tobacco-ban-hurting-pick-n-pay-as-it-counts-the-cost-of-covid-19/
25	2020-06-05	Business Tech	Staff Reporter	South Africa’s cigarette ban heads to court next week	https://businesstech.co.za/news/business/405345/south-africas-cigarette-ban-heads-to-court-next-week/
26	2020-06-30	Business Tech	Staff Reporter	South Africa’s cigarette ban backfire	https://businesstech.co.za/news/finance/421096/south-africas-cigarette-ban-backfire/

27	2020-08-04	Daily Maverick	Narnia Bohler-Muller, Stephen Rule, Martin Bekker & Ben Roberts	Smoke and mirrors? Public perceptions on banning the sale of cigarettes	https://www.dailymaverick.co.za/article/2020-08-04-smoke-and-mirrors-public-perceptions-on-banning-the-sale-of-cigarettes/
28	2020-04-09	Daily Maverick	Savera Kalideen, Sharon Nyatsanza, Pamela Naidoo, Lorraine Govender and Catherine Egbe	Covid-19 Smoking Ban: The good, the bad and the very ugly	https://www.dailymaverick.co.za/article/2020-04-09-covid-19-smoking-ban-the-good-the-bad-and-the-very-ugly/
29	2020-10-07	Daily Maverick	Savera Kalideen and Sharon Nyatsanza	The mini budget is the ideal opportunity to whack up tobacco taxes – lockdown has shown us the way	https://www.dailymaverick.co.za/article/2020-10-21-the-mini-budget-is-the-ideal-opportunity-to-whack-up-tobacco-taxes-lockdown-has-shown-us-the-way/
30	2020-08-15	Daily Maverick	Ferial Haffajee	Ramaphosa takes South Africa out of hard lockdown, lifts cigarette and alcohol ban	https://www.dailymaverick.co.za/article/2020-08-15-ramaphosa-takes-south-africa-out-of-hard-lockdown-lifts-cigarette-and-alcohol-ban/
31	2020-05-11	Daily Maverick	Melinda Ferguson	How I nearly sued the government to allow me to smoke	https://www.dailymaverick.co.za/article/2020-05-11-how-i-nearly-sued-the-government-to-allow-me-to-smoke/
32	2020-05-29	Daily Maverick	Ferial Haffajee	Dlamini Zuma's tobacco ban and the political divisions it is causing	https://www.dailymaverick.co.za/article/2020-05-29-dlamini-zumas-tobacco-ban-and-the-political-divisions-it-is-causing/
33	2020-05-28	Daily Maverick	Centre for Analytics and Behavioural Change	South Africa's 24-hour trend report for 29 April 2020	https://www.dailymaverick.co.za/article/2020-04-30-south-africas-24-hour-trend-report-for-29-april-2020/

34	2020-04-09	Daily Maverick	Des Erasmus	Even presidents can change their minds, says Ramaphosa about cigarette U-turn	https://www.dailymaverick.co.za/article/2020-05-05-even-presidents-can-change-their-minds-says-ramaphosa-about-cigarette-u-turn/
35	2020-06-26	Daily Maverick	Vanessa Burger	Coalescence of RET forces behind cigarette ban suggests more at stake than 'nation's health	https://www.dailymaverick.co.za/opinionista/2020-05-25-coalescence-of-ret-forces-behind-cigarette-ban-suggests-more-at-stake-than-nations-health/
36	2020-09-27	Daily Maverick	Sandisiwe Shoba	South Africans raise their concerns directly with President Ramaphosa	https://www.dailymaverick.co.za/article/2020-07-02-south-africans-raise-their-concerns-directly-with-president-ramaphosa/
37	2020-04-30	Daily Maverick	Maurice Smithers	Alcohol, alcohol harm and alcohol dependency: Putting things in perspective	https://www.dailymaverick.co.za/article/2020-04-13-alcohol-alcohol-harm-and-alcohol-dependency-putting-things-in-perspective/
38	2020-05-25	Daily Maverick	Stephen Grootes	Government's strange silence in the tobacco war of its own making	https://www.dailymaverick.co.za/article/2020-06-09-governments-strange-silence-in-the-tobacco-war-of-its-own-making/
39	2020-05-04	Daily Maverick	Maurice Smithers	Minimising health risks under Covid	https://www.dailymaverick.co.za/article/2020-05-27-more-must-be-done-to-limit-alcohol-access-as-is-case-with-tobacco/
40	2020-07-02	Daily Maverick	Centre for Analytics and Behavioural Change	South Africa's 24-hour trend report — 13 April 2020	https://www.dailymaverick.co.za/article/2020-04-13-south-africas-24-hour-trend-report-13-april-2020/
41	2020-04-13	Daily Maverick	Ed Stoddard	UCT study finds soaring prices for illicit cigarettes burn smokers, suggests sin tax hike	https://www.dailymaverick.co.za/article/2020-07-21-uct-study-finds-soaring-prices-for-illicit-cigarettes-burn-smokers-suggests-sin-tax-hike/
42	2020-06-09	Daily Maverick	Matthew van der Want	Tobacco ban: Try to explain 'rational' to an addict	https://www.dailymaverick.co.za/opinionista/2020-06-28-tobacco-ban-try-to-explain-rational-to-an-addict/

43	2020-05-27	Daily Maverick	Ed Stoddard	Pick n Pay flags above-50% interim profit fall, cites alcohol and tobacco bans	https://www.dailymaverick.co.za/article/2020-08-04-pick-n-pay-flags-above-50-interim-profit-fall-cites-alcohol-and-tobacco-bans/
44	2020-04-13	Daily Maverick	Zacharia Motsumi	Covid-19 gave criminal syndicates control of the tobacco market – let's take back control	https://www.dailymaverick.co.za/opinionista/2020-10-27-covid-19-gave-criminal-syndicates-control-of-the-tobacco-market-lets-take-back-control/
45	2020-07-21	Daily Maverick	Greg Nicolson	As restrictions ease, Nkosazana Dlamini Zuma urges vigilance against second wave of Covid-19	https://www.dailymaverick.co.za/article/2020-08-18-as-restrictions-ease-nkosazana-dlamini-zuma-urges-vigilance-against-second-wave-of-covid-19/
46	2020-08-04	Daily Maverick	Tebele Luthuli	Illicit trade in cigarettes and alcohol has thrived during lockdown	https://www.dailymaverick.co.za/opinionista/2020-06-05-illicit-trade-in-cigarettes-and-alcohol-has-thrived-during-lockdown/
47	2020-06-28	Daily Maverick	Centre for Analytics and Behavioural Change	South Africa's 24-hour trend report – 4 May 2020	https://www.dailymaverick.co.za/article/2020-05-05-south-africas-24-hour-trend-report-4-may-2020/
48	2020-08-26	Daily Maverick	Cyril Ramaphosa	Reconsidering the position on tobacco was a collective decision	https://www.dailymaverick.co.za/article/2020-05-04-reconsidering-the-position-on-tobacco-was-a-collective-decision/
49	2020-08-04	Daily Maverick	Centre for Analytics and Behavioural Chang	South Africa's 24-hour trend report – the weekend	https://www.dailymaverick.co.za/article/2020-05-25-south-africas-24-hour-trend-report-the-weekend/
50	2020-08-18	Daily Maverick	Karabo Mafolo	Sixty percent of lockdown arrests in Cape Town were shopkeepers selling alcohol and cigarettes	https://www.dailymaverick.co.za/article/2020-04-24-sixty-percent-of-lockdown-arrests-in-cape-town-were-shopkeepers-selling-alcohol-and-cigarettes/
51	2020-06-05	Daily Maverick	Terry Bell	Smoke and memories	https://www.dailymaverick.co.za/opinionista/2020-04-23-smoke-and-memories/

52	2020-09-11	Daily Maverick	Sharon Nyatsanza	Cigarette butts stink – and are a major cause of plastic pollution	https://www.dailymaverick.co.za/opinionista/2020-07-30-cigarette-butts-stink-and-are-a-major-cause-of-plastic-pollution/
53	2020-05-05	Daily Maverick	Sisi Ntombela and Meokgo Matuba	This is why the tobacco ban must stay	https://www.dailymaverick.co.za/opinionista/2020-05-29-this-is-why-the-tobacco-ban-must-stay/
54	2020-05-04	Daily Maverick	Allen Gallagher, Mateusz Zatonski, Tom Hird and Lekan Ayo-Yusuf	The tobacco industry's hypocrisy on illicit trade	https://www.dailymaverick.co.za/article/2020-07-09-the-tobacco-industrys-hypocrisy-on-illicit-trade/
55	2020-07-30	Daily Maverick	Christi Nortier	Big tobacco heads to court over sale ban, Denel sags under pandemic pressure and Level 3 conundrum for childcare	https://www.dailymaverick.co.za/article/2020-05-29-big-tobacco-heads-to-court-over-sale-ban-denel-sags-under-pandemic-pressure-and-level-3-conundrum-for-childcare/
56	2020-07-30	Daily Maverick	Marianne Merten	Alcohol and tobacco ban costs SA's collapsing economy an additional R1.7bn	https://www.dailymaverick.co.za/article/2020-05-01-alcohol-and-tobacco-ban-costs-sas-collapsing-economy-an-additional-r1-7bn/
57	2020-05-29	Daily Maverick	Rebecca Davis	Unpacking the rationality of South Africa's lockdown regulations	https://www.dailymaverick.co.za/article/2020-05-14-unpacking-the-rationality-of-south-africas-lockdown-regulations/
58	2020-07-09	Daily Maverick	Lucas Ledwaba	A hazy future for some farmers despite tobacco ban lift	https://www.dailymaverick.co.za/article/2020-08-18-a-hazy-future-for-some-farmers-despite-tobacco-ban-lift-2/
59	2020-04-21	Daily Maverick	Kopo Mapila	Lifting the veil: A philosophical investigation of the tobacco ban	https://www.dailymaverick.co.za/opinionista/2020-06-10-lifting-the-veil-a-philosophical-investigation-of-the-tobacco-ban/
60	2020-05-29	Daily Maverick	Ferial Haffajee	Dlamini Zuma turns cigarettes into illicit drugs as the underground economy takes over	https://www.dailymaverick.co.za/article/2020-06-12-dlamini-zuma-turns-cigarettes-into-illicit-drugs-as-the-underground-economy-takes-over/

61	2020-06-08	Daily Maverick	Mukovhe Morris Masutha	Profit before wellbeing: British American Tobacco vs Nkosazana Dlamini Zuma	https://www.dailymaverick.co.za/opinionista/2020-05-20-profit-before-wellbeing-british-american-tobacco-vs-nkosazana-dlamini-zuma/
62	2020-08-10	Daily Maverick	Daniel Steyn	Should alcohol, tobacco and outdoor exercise be banned?	https://www.dailymaverick.co.za/article/2020-04-17-should-alcohol-tobacco-and-outdoor-exercise-be-banned/
63	2020-05-01	Daily Maverick	Zukiswa Pikoli	Black market cigarettes sales rise, Parliament passes February Budget, and education department hauled to court over school nutrition	https://www.dailymaverick.co.za/article/2020-06-14-black-market-cigarettes-sales-rise-parliament-passes-february-budget-and-education-department-hauled-to-court-over-school-nutrition/
64	2020-05-14	Daily Maverick	Ivor Sarakinsky	Cigarettes and the Smoking Gun	https://www.dailymaverick.co.za/opinionista/2020-05-06-cigarettes-and-the-smoking-gun/
65	2020-06-10	Daily Maverick	David Clement and Martin van Staden	Prohibition and paternalism are always wrong, especially in a pandemic	https://www.dailymaverick.co.za/opinionista/2020-06-16-prohibition-and-paternalism-are-always-wrong-especially-in-a-pandemic/
66	2020-06-12	Daily Maverick	Bianca Coleman	After the mad scramble for booze and cigarettes, reality bites	https://www.dailymaverick.co.za/article/2020-03-27-after-the-mad-scramble-for-booze-and-cigarettes-reality-bites/
67	2020-05-20	Daily Maverick	Professor Balthazar	Covid-19 regulations: Two judgments, two imperfect documents	https://www.dailymaverick.co.za/opinionista/2020-06-29-covid-19-regulations-two-judgments-two-imperfect-documents/
68	2020-04-17	Daily Maverick	Ed Stoddard	Prohibition: Social conservatism that's shattering working-class lives	https://www.dailymaverick.co.za/article/2020-08-10-prohibition-social-conservatism-thats-shattering-working-class-lives/
69	2020-06-14	Daily Maverick	Ferial Haffajee	Prohibition lockdown goes rogue	https://www.dailymaverick.co.za/article/2020-04-20-prohibition-lockdown-goes-rogue/
70	2020-06-16	Daily Maverick	Ferial Haffajee	Ain't no drag getting hold of cigarettes	https://www.dailymaverick.co.za/article/2020-05-09-aint-no-drag-getting-hold-of-cigarettes/

71	2020-03-27	Daily Maverick	Ed Stoddard	Interview: SA’s vaping industry is going up in smoke	https://www.dailymaverick.co.za/article/2020-07-22-interview-sas-vaping-industry-is-going-up-in-smoke/
72	2020-06-29	Daily Maverick	PSG	Jacques Pauw and other industry-experts share their views in PSG’s Think Big webinar series	https://www.dailymaverick.co.za/article/2020-07-08-jacques-pauw-and-other-industry-experts-share-their-views-in-psgs-think-big-webinar-series/
73	2020-08-10	Daily Maverick	Christi Nortier	Still no final word on Level 3 details, SANDF clears soldiers of lockdown killing, and cigarette ban defended in court papers	https://www.dailymaverick.co.za/article/2020-05-27-still-no-final-word-on-level-3-details-sandf-clears-soldiers-of-lockdown-killing-and-cigarette-ban-defended-in-court-papers/
74	2020-04-20	Daily Maverick	Narnia Bohle-Muller	Questions remain over the government’s handling of supposedly vulnerable smokers	https://www.dailymaverick.co.za/article/2020-07-12-questions-remain-over-the-governments-handling-of-supposedly-vulnerable-smokers/
75	2020-04-06	Daily Maverick	Marianne Merten	As South Africa’s capacity teeters under lockdown, the illicit economy costs public purse billions	https://www.dailymaverick.co.za/article/2020-05-06-as-south-africas-capacity-teeters-under-lockdown-the-illicit-economy-costs-public-purse-billions/
76	2020-08-06	Daily Maverick	Adriano Mazzotti	I am not an ‘illicit tobacco kingpin’ and am opposed to the illicit tobacco trade	https://www.dailymaverick.co.za/article/2020-08-11-i-am-not-an-illicit-tobacco-kingpin-and-am-opposed-to-the-illicit-tobacco-trade/
77	2020-05-09	Daily Maverick	John Steenhuisen	Straight Talk: ANC lockdown is based on fear, not facts	https://www.dailymaverick.co.za/opinionista/2020-06-05-straight-talk-anc-lockdown-is-based-on-fear-not-facts/
78	2020-07-22	Daily Maverick	Centre for Analytics and Behavioural Change	South Africa’s 24-hour trend report – 3 May 2020	https://www.dailymaverick.co.za/article/2020-05-04-south-africas-24-hour-trend-report-3-may-2020/

79	2020-07-08	Daily Maverick	Christi Nortier	Witnesses tell of assault on Collins Khosa, arrested waste pickers held without trial, and more South Africans repatriated	https://www.dailymaverick.co.za/article/2020-06-10-witnesses-tell-of-assault-on-collins-khosa-arrested-waste-pickers-held-without-trial-and-more-south-africans-repatriated/
80	2020-05-27	Daily Maverick	Stephen Grootes	Level 3 could be the short-term panacea the government needs right now	https://www.dailymaverick.co.za/article/2020-05-19-level-3-could-be-the-short-term-panacea-the-government-needs-right-now/
81	2020-07-12	Daily Maverick	Omphemetse S Sibanda	We are not alone: US courts are also grappling with challenges to lockdown regulations	https://www.dailymaverick.co.za/opinionista/2020-07-30-we-are-not-alone-us-courts-are-also-grappling-with-challenges-to-lockdown-regulations/
82	2020-05-06	Daily Maverick	Ed Stoddard	Let's add 20% to the sin tax as an incentive to lift the prohibition	https://www.dailymaverick.co.za/article/2020-05-12-lets-add-20-to-the-sin-tax-as-an-incentive-to-lift-the-prohibition/
83	2020-08-11	Daily Maverick	Stephen Grootes	Level 3 – a slightly less terrible option	https://www.dailymaverick.co.za/article/2020-05-26-level-3-a-slightly-less-terrible-option/
84	2020-06-05	Daily Maverick	Centre for Analytics and Behavioural Change	South Africa's 24 hour trend report – 30 April 2020	https://www.dailymaverick.co.za/article/2020-05-01-south-africas-24-hour-trend-report-30-april-2020/
85	2020-05-04	Daily Maverick	Gary Pienaar, Michael Cosser and Narnia Bohler-Muller	Covid-19 and the role of the judiciary: Contradictions and confusion	https://www.dailymaverick.co.za/article/2020-07-15-covid-19-and-the-role-of-the-judiciary-contradictions-and-confusion/
86	2020-06-10	Daily Maverick	Zukiswa Pikoli	Disaster regulations are not open to interpretation, as some ministers seem to think	https://www.dailymaverick.co.za/article/2020-06-03-disaster-regulations-are-not-open-to-interpretation-as-some-ministers-seem-to-think/

87	2020-05-19	Daily Maverick	Centre for Analytics and Behavioural Change	South Africa’s 24-hour trend report – 18 May 2020	https://www.dailymaverick.co.za/article/2020-05-19-south-africas-24-hour-trend-report-18-may-2020/
88	2020-07-30	Daily Maverick	Roger Stewart	Guided by the evidence? The problem is the pandemic, and the evidence, are moving targets	https://www.dailymaverick.co.za/opinionista/2020-06-11-guided-by-the-evidence-the-problem-is-the-pandemic-and-the-evidence-are-moving-targets/
89	2020-05-12	Daily Maverick	Mary de Haas	Learning from lockdown mistakes: The way forward	https://www.dailymaverick.co.za/article/2020-05-22-learning-from-lockdown-mistakes-the-way-forward/
90	2020-05-26	Daily Maverick	Marianne Merten	Ceding control to faceless securocrats and unaccountable governance structures chips away at SA’s constitutional democracy, one broken bit at a time	https://www.dailymaverick.co.za/article/2020-05-25-ceding-control-to-faceless-securocrats-and-unaccountable-governance-structures-chips-away-at-sas-constitutional-democracy-one-broken-bit-at-a-time/
91	2020-05-01	Daily Maverick	J Brooks Spector	Prohibition, the stiffest drink of all	https://www.dailymaverick.co.za/article/2020-04-24-prohibition-the-stiffest-drink-of-all/
92	2020-04-22	eNCA	Staff Reporter	Tobacco association fired-up over cigarette ban	https://www.enca.com/news/tobacco-association-calls-cigarette-ban-be-lifted
93	2020-04-30	eNCA	Staff Reporter	Tobacco association to fight cigarettes ban in court	https://www.enca.com/news/tobacco-association-fight-cigarettes-ban-court
94	2020-08-06	eNCA	Staff Reporter	BATSA burns holes in tobacco ban	https://www.enca.com/news/batsa-says-tobacco-ban-unscientific-court-case
95	2020-05-28	eNCA	Staff Reporter	NCCC submits tobacco ban motivation	https://www.enca.com/analysis/nccc-submits-tobacco-ban-motivation
96	2020-05-12	eNCA	Staff Reporter	HSRC: Most citizens sticking to tobacco ban	https://www.enca.com/news/most-sa-adhere-lockdown-regulations-around-banned-tobacco
97	2020-07-03	eNCA	Staff Reporter	Tobacco ban: Fita files application for leave to appeal	https://www.enca.com/news/tobacco-ban-fita-files-application-leave-appeal

98	2020-07-22	eNCA	Staff Reporter	Govt's alcohol, tobacco ban tweet befuddles SA	https://www.enca.com/news/Govt-alcohol-tobacco-ban-tweet-befudles-SA
99	2020-07-09	eNCA	Staff Reporter	Tobacco ban appeal to be heard next week	https://www.enca.com/news/tobacco-ban-appeal-be-heard-next-week
100	2020-06-10	eNCA	Staff Reporter	SA Lockdown: Tobacco battle in court	https://www.enca.com/news/sa-lockdown-tobacco-battle-court
101	2020-07-15	eNCA	Staff Reporter	Fita asks for leave to appeal tobacco ban	https://www.enca.com/news/fita-asks-leave-appeal-tobacco-ban
102	2020-06-05	eNCA	Staff Reporter	No postponement of tobacco challenge	https://www.enca.com/news/no-postponement-tobacco-challenge
103	2020-05-12	eNCA	Staff Reporter	SA Lockdown: Court battle over tobacco ban on hold	https://www.enca.com/news/court-battle-over-tobacco-ban-hold
104	2020-05-11	eNCA	Staff Reporter	Tobacco association heads to court to fight ban	https://www.enca.com/news/tobacco-heads-court-fight-ban
105	2020-04-13	eNCA	Staff Reporter	Calls to lift cigarette sales ban	https://www.enca.com/news/watch-calls-lift-cigarette-sales-ban
106	2020-05-04	eNCA	Staff Reporter	Ramaphosa part of decision to ban sale of tobacco products	https://www.enca.com/news/ramaphosa-part-decision-ban-sale-tobacco-products
107	2020-05-06	eNCA	Staff Reporter	BAT won't pursue court action against cigarettes ban	https://www.enca.com/news/bat-wont-pursue-court-action-against-cigarettes-ban
108	2020-05-31	eNCA	Staff Reporter	Ramaphosa: I should've announced the re-banning of cigarettes	https://www.enca.com/news/ramaphosa-i-shouldve-announced-re-banning-cigarettes
109	2020-08-26	eNCA	Staff Reporter	Fita wants govt engagement should cigarette ban return	https://www.enca.com/news/fita-wants-govt-engagement-should-cigarette-ban-return
110	2020-08-26	eNCA	Staff Reporter	Fita settles tobacco court case with government	https://www.enca.com/news/fita-settles-tobacco-court-case-govt
111	2020-08-01	eNCA	Staff Reporter	Fita heads to Supreme Court of Appeal over tobacco ban	https://www.enca.com/news/fita-heads-supreme-court-appeal-over-tobacco-ban
112	2020-04-18	eNCA	Staff Reporter	Tobacco association taking government to court	https://www.enca.com/news/tobacco-association-taking-government-court

113	2020-05-29	eNCA	Staff Reporter	Cele: Proof of cigarette purchases required	https://www.enca.com/news/cele-doubles-down-smoking-comments
114	2020-05-04	eNCA	Staff Reporter	British American Tobacco sets deadline for government to revoke ban	https://www.enca.com/news/british-american-tobacco-sets-deadline-government-revoke-ban
115	2020-08-07	eNCA	Staff Reporter	Cigarette shipment to rehab centre justified: Lukhele	https://www.enca.com/news/cigarette-shipment-rehab-centre-justified-lukhele
116	2020-08-07	eNCA	Staff Reporter	SA lockdown: Tobacco industry fired up and ready to roll	https://www.enca.com/news/sa-lockdown-tobacco-industry-prepares-get-things-rolling
117	2020-08-07	Eye Witness News	Kaylynn Palm	Judgment in tobacco sale ban matter between BATSA, COGTA has been reserved	https://ewn.co.za/2020/08/07/judgment-in-tobacco-sale-ban-between-british-american-tobacco-cogta-reserved
118	2020-08-05	Eye Witness News	Kaylynn Palm	British American Tobacco's cigarette sale ban bid to be heard in court	https://ewn.co.za/2020/08/05/british-american-tobacco-s-cigarette-sale-ban-bid-to-be-heard-in-court
119	2020-06-01	Eye Witness News	Clement Manyathela	Ramaphosa admits he should have explained govt's U-turn on cigarette sales	https://ewn.co.za/2020/06/01/ramaphosa-admits-he-should-have-explained-govt-s-u-turn-on-cigarette-sales
120	2020-05-03	Eye Witness News	Ayanda Nyati	ANCWL: Attack on Dlamini-Zuma over tobacco ban aims to pit her against Ramaphosa	https://ewn.co.za/2020/05/03/ancwl-attack-on-dlamini-zuma-over-tobacco-ban-aims-to-pit-her-against-ramaphosa
121	2020-04-30	Eye Witness News	AFP	South Africa's cigarette ban lights up a row	https://ewn.co.za/2020/04/30/south-africa-s-cigarette-ban-lights-up-a-row
122	2020-08-12	Eye Witness News	Kaylynn Palm	Industry players mum on reports of lifting of tobacco, alcohol sale bans	https://ewn.co.za/2020/08/12/industry-players-mum-on-reports-of-lifting-of-tobacco-alcohol-sale-bans
123	2020-08-10	Eye Witness News	Staff Reporter	Too soon to tell if govt winning war against illegal cigarettes - ISS	https://www.enca.com/news/too-soon-tell-if-govt-winning-war-against-illegal-cigarettes-iss

124	2020-08-18	Eye Witness News	Kaylynn Palm	Smokers delighted to be able to buy favourite cigarette brands again	https://ewn.co.za/2020/08/18/smokers-delighted-to-be-able-to-buy-favourite-cigarette-brands-again
125	2020-08-18	Eye Witness News	Kaylynn Palm	Calls for stricter controls after booze and tobacco sale bans lifted	https://ewn.co.za/2020/08/18/limit-adverts-calls-for-stricter-controls-after-booze-and-tobacco-bans-lifted
126	2020-08-18	Eye Witness News	Theto Mahlakoana	I don’t tell Cabinet what to do – NDZ responds to criticism over lockdown bans	https://ewn.co.za/2020/08/18/i-don-t-tell-cabinet-what-to-do-ndz-responds-to-ban-criticism
127	2020-08-18	Eye Witness News	Lauren Isaacs	Don’t panic buy! PnP welcomes liquor and cigarette buyers	https://ewn.co.za/2020/08/18/don-t-panic-buy-pnp-welcomes-liquor-and-cigarette-buyers
128	2020-08-18	Eye Witness News	Veronica Mokhoali	Money's no issue: R5k bill & more as shoppers queue to stock up after booze ban	https://ewn.co.za/2020/08/18/r5k-liquor-bill-and-more-as-shoppers-queue-to-stock-up-after-ban
129	2020-08-18	Eye Witness News	Veronica Mokhoali	Bottoms up! Long queues outside liquor stores in Joburg already	https://ewn.co.za/2020/08/18/long-queues-already-begin-outside-liquor-stores-in-johannesburg
130	2020-08-18	Eye Witness News	Veronica Mokhoali	Some S. Africans show cautious optimism over eased rules under alert level 2	https://ewn.co.za/2020/08/18/sa-moves-to-alert-level-2-some-citizens-show-cautious-optimism-over-eased-rules
131	2020-08-17	Eye Witness News	Kayleen Morgan	Tobacco, alcohol, inter-provincial travel & curfew - level 2 lockdown amendments	https://ewn.co.za/video/11787/tobacco--alcohol--inter-provincial-travel---curfew----level-2-lockdown-amendments
132	2020-08-17	Eye Witness News	Staff Reporter	Rand slips as stock market gains on easing lockdown rules	https://ewn.co.za/2020/08/17/rand-slips-as-stock-market-gains-on-easing-lockdown-rules
133	2020-08-17	Eye Witness News	Veronica Mokhoali	Safety in spotlight as retailers prepare for return of alcohol, cigarettes sales	https://ewn.co.za/2020/08/17/safety-in-spotlight-as-retailers-prepare-for-return-of-alcohol-cigarettes-sales

134	2020-08-17	Eye Witness News	Thapelo Lekabe	Dlamini-Zuma hits out at those blaming her for tough lockdown regulations	https://ewn.co.za/2020/08/17/dlamini-zuma-hits-out-at-those-blaming-her-for-tough-lockdown-regulations
135	2020-08-17	Eye Witness News	Staff Reporter	No specific regulations on sale of tobacco products, says Dlamini-Zuma	https://ewn.co.za/2020/08/17/watch-live-nccc-ministers-brief-media-on
136	2020-08-16	Eye Witness News	Kevin Brandt	DA slams international travel ban, 10pm curfew & limited school attendance	https://ewn.co.za/2020/08/16/da-slams-international-travel-ban-10pm-curfew-and-limited-school-attendance
137	2020-08-16	Eye Witness News	Ayanda Nyathi	Political parties react; EFF rejects easing of lockdown regulations	https://ewn.co.za/2020/08/16/political-parties-react-eff-rejects-easing-of-lockdown-regulations
138	2020-08-16	Eye Witness News	Eduard De Kock & Ayanda Nyathi	As restaurants get ready for recovery, Fita to continue tobacco legal battle	https://ewn.co.za/2020/08/16/as-restaurants-get-ready-for-recovery-fita-to-continue-tobacco-legal-battle
139	2020-08-16	Eye Witness News	Ray White	'Lifting of sales ban on alcohol, cigarettes long overdue'	https://ewn.co.za/2020/08/16/lifting-of-ban-on-alcohol-cigarettes-long-overdue
140	2020-08-15	Eye Witness News	Staff Reporter	Booze & cigarette sales unbanned	https://ewn.co.za/live/LIVE-BLOG--Will-booze-and-cigarette-sales-be-unbanned-Ramaphosa-addresses-the-nation
141	2020-08-15	Eye Witness News	Staff Reporter	FULL SPEECH: President Cyril Ramaphosa announces level 2 lockdown	https://ewn.co.za/2020/08/15/full-speech-president-cyril-ramaphosa-announces-level-2-lockdown
142	2020-08-15	Eye Witness News	Shimoney Regter	SA lockdown: Ramaphosa to address the nation tonight	https://ewn.co.za/2020/08/15/sa-lockdown-ramaphosa-to-address-the-nation-tonight
143	2020-08-15	Eye Witness News	Sifiso Zulu	SCA grants Fita leave to appeal dismissal of tobacco sales ban	https://ewn.co.za/2020/08/15/sca-grants-fita-leave-to-appeal-dismissal-of-tobacco-sales-ban

144	2020-08-15	Eye Witness News	Kaylynn Palm	City of CT ready for talks to ease lockdown, unban sale of booze and cigarettes	https://ewn.co.za/2020/08/15/city-of-ct-ready-for-talks-to-ease-lockdown-unban-booze-and-cigarettes
145	2020-08-13	Eye Witness News	Mia Lindeque	Cosatu hopes Ramaphosa will soon announce reopening of more sectors of economy	https://ewn.co.za/2020/08/13/cosatu-hopes-ramaphosa-will-soon-announce-reopening-of-more-sectors-of-economy
146	2020-08-12	Eye Witness News	Bonga Dlulane	Cosatu calls for alcohol sales ban to be lifted, says black market thriving	https://ewn.co.za/2020/08/12/cosatu-calls-for-alcohol-sales-ban-to-be-lifted-says-black-market-thriving
147	2020-08-12	Eye Witness News	Lauren Isaacs	Paarl police make tobacco bust of products worth more than R1 million	https://ewn.co.za/2020/08/12/paarl-police-make-tobacco-bust-of-products-worth-more-than-r1-million
148	2020-08-12	Eye Witness News	Nkosikhona Duma	DA's Steenhuisen: Ramaphosa must end hard lockdown now	https://ewn.co.za/2020/08/12/john-steenhuisen-it-s-time-to-rebuild-economy-end-hard-lockdown
149	2020-08-11	Eye Witness News	Nkosikhona Duma	Cosatu backs calls to lift ban on alcohol sales, but with conditions	https://ewn.co.za/2020/08/11/cosatu-backs-calls-to-lift-ban-on-alcohol-sales-but-with-conditions
150	2020-08-07	Eye Witness News	Kaylynn Palm	While waiting on court ruling, Batsa vows to fight illicit cigarette trade	https://ewn.co.za/2020/08/07/while-waiting-on-court-ruling-batsa-vows-to-keep-fighting-illicit-cigarette-trade
151	2020-08-06	Eye Witness News	Nkosikhona Duma	Mkhize: SA not ready to lift ban on sales of tobacco, alcohol yet	https://ewn.co.za/2020/08/06/mkhize-sa-not-ready-to-lift-ban-on-sale-of-tobacco-alcohol-yet
152	2020-08-06	Eye Witness News	Kaylynn Palm	Dlamini-Zuma has a duty to prevent spread of COVID-19 & save lives, court told	https://ewn.co.za/2020/08/06/dlamini-zuma-has-a-duty-to-prevent-spread-of-covid-19-and-save-lives-court-told
153	2020-08-06	Eye Witness News	Eyewitness News	Liquor, cigarette ban in place to protect lives in pandemic–Dlamini-Zuma lawyer	https://ewn.co.za/2020/08/06/liquor-cigarette-ban-in-place-to-save-protect-lives-in-pandemic-dlamini-zuma-lawyer

154	2020-08-06	Eye Witness News	Lauren Isaacs	Farmers on the brink of ruin due to alcohol and tobacco ban: SAAI	https://ewn.co.za/2020/08/06/famers-on-the-brink-of-ruin-due-to-alcohol-and-tobacco-ban-saai
155	2020-08-06	Eye Witness News	Regan Thaw & Veronica Mokhoali	BLSA: Govt must consider economy's recovery when deciding on alcohol,tobacco ban	https://ewn.co.za/2020/08/06/blsa-govt-must-consider-economy-s-recovery-when-deciding-on-alcohol-tobacco-ban
156	2020-08-05	Eye Witness News	Veronica Mokhoali	BLSA backs call to lift bans on sale of alcohol, cigarettes	https://ewn.co.za/2020/08/05/blsa-backs-call-to-lift-bans-on-sale-of-alcohol-cigarettes
157	2020-08-05	Eye Witness News	Kaylynn Palm	Tobacco sales ban violates consumer rights, free trade rights - Batsa	https://ewn.co.za/2020/08/05/tobacco-sales-ban-violates-consumer-rights-free-trade-rights-batsa
158	2020-08-05	Eye Witness News	Judith February	JUDITH FEBRUARY: Nothing about democratic progress is inevitable	https://ewn.co.za/2020/08/05/judith-february-nothing-about-democratic-progress-is-inevitable
159	2020-08-05	Eye Witness News	Regan Thaw & Kaylynn Palm	British American Tobacco argues in court why cigarette sale ban must be scrapped	https://ewn.co.za/2020/08/05/british-american-tobacco-argues-in-court-why-cigarette-sale-ban-must-be-scrapped
160	2020-08-05	Eye Witness News	Staff Reporter	Cost of tobacco ban outweighs benefit'-Industry challenges ban in WC High Court	https://ewn.co.za/2020/08/05/cost-of-tobacco-ban-outweighs-benefit-industry-challenges-ban-in-wc-high-court
161	2020-08-04	Eye Witness News	Bonga Dlulane	SCA agrees to urgently hear Fita appeal bid in cigarette sales ban matter	https://ewn.co.za/2020/08/04/sca-agrees-to-urgently-hear-fita-appeal-bid-in-cigarette-sales-ban-matter
162	2020-08-04	Eye Witness News	Nkosikhona Duma	The DA wants alcohol, tobacco ban lifted with immediate effect	https://ewn.co.za/2020/08/04/the-da-wants-alcohol-tobacco-ban-lifted-with-immediate-effect
163	2020-07-30	Eye Witness News	Jarita Kassen	Seasonal farm worker jobs expected to be worst-hit by COVID-19 pandemic	https://ewn.co.za/2020/07/30/seasonal-farm-worker-jobs-expected-to-be-worst-hit-by-covid-19-pandemic

164	2020-07-29	Eye Witness News	Jarita Kassen	Agriculture sector has weathered COVID-19 storm, says chief economist	https://ewn.co.za/2020/07/29/agriculture-sector-has-weathered-covid-19-storm-says-chief-economist
165	2020-07-25	Eye Witness News	Ayanda Nyathi	Tobacco sales ban: Fita to petition SCA as early as next week	https://ewn.co.za/2020/07/25/tobacco-sales-ban-fita-to-petition-sca-as-early-as-next-week
166	2020-07-24	Eye Witness News	Ayanda Nyathi	Fita: We're confident that SCA will overturn tobacco sales ban	https://ewn.co.za/2020/07/24/fita-we-re-confident-the-sca-will-overturn-tobacco-sales-ban
167	2020-07-23	Eye Witness News	Regan Thaw	SA tobacco market completely taken over by illicit suppliers - Batsa	https://ewn.co.za/2020/07/23/sa-tobacco-market-completely-taken-over-by-illicit-suppliers-batsa
168	2020-07-22	Eye Witness News	Reuters Editorial	SA's retail sales plunge in April and May after hard lockdown	https://ewn.co.za/2020/07/22/sa-s-retail-sales-plunge-in-april-and-may-after-hard-lockdown
169	2020-07-22	Eye Witness News	Lungelo Matangira	Govt backtracks on 'incorrect' tweet on booze & cigarette bans during lockdown	https://ewn.co.za/2020/07/22/govt-backtracks-on-incorrect-tweet-on-booze-and-cigarette-bans-during-lockdown
170	2020-07-22	Eye Witness News	Lungelo Matangira	Booze & cigarette bans to stay throughout lockdown - government	https://ewn.co.za/2020/07/22/update-government-clarifies-tweet-on-booze-and-cigarette-bans-during-lockdown
171	2020-07-22	Eye Witness News	Kaylynn Palm	Sharing of cigarettes jumped 430% during lockdown, study finds	https://ewn.co.za/2020/07/22/sharing-of-cigarettes-jumped-430-during-lockdown-study-finds
172	2020-07-21	Eye Witness News	Lungelo Matangira	Study: Smokers try to quit as illegal cigarette prices surge 250% under lockdown	https://ewn.co.za/2020/07/21/study-smokers-try-to-quit-as-illegal-cigarette-prices-up-by-250-under-lockdown
173	2020-07-18	Eye Witness News	Shamiela Fisher	EC man's suspicious behaviour got him caught with liquid tobacco worth R360k	https://ewn.co.za/2020/07/18/ec-man-s-suspicious-behaviour-got-him-caught-with-liquid-tobacco-worth-r360k

174	2020-07-17	Eye Witness News	Staff Reporter	Kabega Park police, in EC, discover liquid tobacco worth around R360k	https://ewn.co.za/2020/07/17/kabega-park-police-in-ec-discover-liquid-tobacco-worth-around-r360k
175	2020-07-16	Eye Witness News	Theto Mahlakoana	Fita accused to challenging cigarette ban to protect monetary gains	https://ewn.co.za/2020/07/16/fita-accused-to-challenging-cigarette-ban-to-protect-monetary-gains
176	2020-07-15	Eye Witness News	Theto Mahlakoana	Fita: We believe Supreme Court of Appeal will rule in favour of tobacco sale bid	https://ewn.co.za/2020/07/15/fita-believes-supreme-court-of-appeal-will-rule-in-its-fire-in-tobacco-sale-bid
177	2020-07-15	Eye Witness News	Theto Mahlakoana	Lawyer: Dlamini-Zuma looked at economic, psychological impact of tobacco ban	https://ewn.co.za/2020/07/15/lawyer-dlamini-zuma-looked-at-economic-psychological-impact-of-tobacco-ban
178	2020-07-15	Eye Witness News	Shimoney Regter	Expect more roadblocks and patrols, warns Cele	https://ewn.co.za/2020/07/15/expect-more-roadblocks-and-patrols-warns-cele
179	2020-07-15	Eye Witness News	Theto Mahlakoana	Fita back in court to appeal dismissal of tobacco case against govt	https://ewn.co.za/2020/07/15/fita-back-in-court-to-appeal-dismissal-of-tobacco-case-against-govt
180	2020-07-10	Eye Witness News	Bonga Dlulane	Fita confident tobacco ban will go up in smoke after appeal bid approved	https://ewn.co.za/2020/07/10/fita-confident-tobacco-ban-will-go-up-in-smoke-after-appeal-bid-approved
181	2020-07-09	Eye Witness News	Bonga Dlulane	High Court grants Fita permission to appeal tobacco sales ban	https://ewn.co.za/2020/07/09/high-court-grants-fita-permission-to-appeal-tobacco-sales-ban
182	2020-07-06	Eye Witness News	Ray White	Stricter lockdown rules not on cards for Gauteng - provincial govt	https://ewn.co.za/2020/07/06/stricter-lockdown-rules-not-on-cards-for-gauteng-provincial-govt
183	2020-07-04	Eye Witness News	Ayanda Nyathi	Fita: Cigarette ban enriching criminals as illicit trade booms	https://ewn.co.za/2020/07/04/fita-cigarette-ban-enriching-criminals-as-illicit-trade-booms

184	2020-07-03	Eye Witness News	Ayanda Nyathi	Here's why Fita’s appealing court decision on tobacco sales ban	https://ewn.co.za/2020/07/03/here-s-why-fita-s-appealing-court-decision-on-tobacco-sales-ban
185	2020-07-03	Eye Witness News	Mia Lindeque	Cop found with illicit cigarettes out on bail	https://ewn.co.za/2020/07/03/cop-found-with-illicit-cigarettes-out-on-bail
186	2020-07-02	Eye Witness News	Jarita Kassen	Illicit tobacco trade 'booming since Sars disbanded its probing capacity'	https://ewn.co.za/2020/07/02/illicit-tobacco-trade-booming-since-sars-disbanded-its-probing-capacity-expert
187	2020-07-01	Eye Witness News	Jarita Kassen	Tobacco firms complicit in illicit cigarette trade, claims expert	https://ewn.co.za/2020/07/01/tobacco-firms-complicit-in-illicit-cigarette-trade-claims-expert
188	2020-06-28	Eye Witness News	Bonga Dlulane	Fita's legal battle with govt over cigarette sale ban may be far from over	https://ewn.co.za/2020/06/28/fita-s-legal-battle-with-govt-over-cigarette-sale-ban-may-be-far-from-over
189	2020-06-27	Eye Witness News	Bonga Dlulane	Government feels vindicated by court ruling keeping cigarette ban in place	https://ewn.co.za/2020/06/27/government-feels-vindicated-by-court-ruling-keeping-cigarette-ban-in-place
190	2020-06-27	Eye Witness News	Veronica Mokhoali	Govt concerned by Batsa claim that it delayed court hearing on tobacco sales ban	https://ewn.co.za/2020/06/27/govt-concerned-by-batsa-accusation-that-it-delayed-court-hearing-on-tobacco-sales-ban
191	2020-06-27	Eye Witness News	Theto Mahlakoana	Govt acted within its powers to ban sale of tobacco products - court	https://ewn.co.za/2020/06/27/govt-acted-within-its-powers-to-ban-sale-of-tobacco-products-court
192	2020-06-26	Eye Witness News	Theto Mahlakoana	Up in smoke: Fita fails in court bid to reinstate sale of cigarettes	https://ewn.co.za/2020/06/26/up-in-smoke-fita-fails-in-court-bid-to-reinstate-sale-of-cigarettes
193	2020-06-26	Eye Witness News	Haji Mohamed Dawjee	HAJI MOHAMED DAWJEE: Cut the cigarette ban already	https://ewn.co.za/2020/06/26/haji-mohamed-dawjee-cut-the-cigarette-ban-already

194	2020-06-14	Eye Witness News	Kaylynn Palm	‘When people zol’ Smokers pushing for tobacco sale say they’ve turned to zolling	https://ewn.co.za/2020/06/14/when-people-zol-capetonians-pushing-for-tobacco-sales-say-they-ve-turned-to-zolling
195	2020-06-13	Eye Witness News	Kaylynn Palm	CT smokers reiterate call for govt to lift ban on tobacco products	https://ewn.co.za/2020/06/13/ct-smokers-reiterate-call-for-govt-to-lift-ban-on-tobacco-products
196	2020-06-12	Eye Witness News	Judith February	JUDITH FEBRUARY: We can’t afford to take our foot off the pedal with COVID-19	https://ewn.co.za/2020/06/12/judith-february-we-can-t-afford-to-take-our-foot-off-the-pedal-in-covid-19-fight
197	2020-06-11	Eye Witness News	Theto Mahlakoana	Govt pleads with court to refer cigarette sale ban matter back to Dlamini-Zuma	https://ewn.co.za/2020/06/11/govt-pleads-with-court-to-refer-cigarette-ban-sale-matter-back-to-dlamini-zuma
198	2020-06-10	Eye Witness News	Theto Mahlakoana	Govt: Tobacco ban may have eased burden on health system	https://ewn.co.za/2020/06/10/govt-tobacco-ban-may-have-eased-burden-on-health-system
199	2020-06-10	Eye Witness News	Staff Reporter	Govt decision to ban sale of cigarettes irrational, Fita tells court	https://ewn.co.za/2020/06/10/govt-decision-to-ban-sale-of-cigarettes-irrational-fita-tells-court
200	2020-06-10	Eye Witness News	Mia Lindeque	Cele ‘concerned’ with spike in murder rate under level 3 lockdown	https://ewn.co.za/2020/06/10/cele-concerned-with-spike-in-murder-rate-under-level-3-lockdown
201	2020-06-09	Eye Witness News	Lawrence Hamilton	ANALYSIS: What sets good and bad leaders apart in coronavirus era	https://ewn.co.za/2020/06/09/analysis-what-sets-good-and-bad-leaders-apart-in-coronavirus-era
202	2020-06-09	Eye Witness News	Theto Mahlakoana	Pretoria High Court to hear Fita’s bid against ban on cigarette sales	https://ewn.co.za/2020/06/09/pretoria-high-court-to-hear-fita-s-bid-against-ban-on-cigarette-sales
203	2020-06-05	Eye Witness News	Nthakoana Ngatane	ANCWL defends Dlamini-Zuma on tobacco decision, wants alcohol ban back	https://ewn.co.za/2020/06/05/ancwl-defends-dlamini-zuma-on-tobacco-decision-wants-alcohol-ban-back

204	2020-06-03	Eye Witness News	Kaylynn Palm	The burning issue: Smokers call for ban on tobacco sales to be lifted	https://ewn.co.za/2020/06/03/smokers-call-for-ban-on-tobacco-sales-to-be-lifted
205	2020-06-02	Eye Witness News	Bonga Dlulane	DA wants Ramaphosa to discipline Dlamini-Zuma over cigarette sales ban	https://ewn.co.za/2020/06/02/da-wants-ramaphosa-to-discipline-dlamini-zuma-over-cigarette-sales-ban
206	2020-06-02	Eye Witness News	Kaylynn Palm	Fired up: Smokers take protest against cigarette sales ban to Parliament	https://ewn.co.za/2020/06/02/fired-up-smokers-take-protest-against-cigarette-sales-ban-to-parliament
207	2020-05-29	Eye Witness News	Staff Reporter	South Africans quench thirst with moonshine during lockdown ban	https://ewn.co.za/2020/05/29/south-africans-quench-thirst-with-moonshine-during-lockdown-ban
208	2020-05-29	Eye Witness News	Staff Reporter	It’s lit now! British American Tobacco heads to court over cigarette sale ban	https://ewn.co.za/2020/05/29/it-s-lit-now-british-american-tobacco-heads-to-court-over-cigarette-sale-ban
209	2020-05-28	Eye Witness News	Clement Manyathela & Theto Mahlakoana	Mthembu: Cigarettes sales will be allowed under level 2	https://ewn.co.za/2020/05/28/mthembu-cigarettes-sales-will-be-allowed-under-level-2
210	2020-05-28	Eye Witness News	Kaylynn Palm	With alcohol sales allowed from 1 June, experts warn of surge in trauma cases	https://ewn.co.za/2020/05/28/with-alcohol-sales-allowed-from-monday-experts-warn-of-surge-in-trauma-cases
211	2020-05-28	Eye Witness News	Theto Mahlakoana	Ban on tobacco sales: Inside the govt’s court papers	https://ewn.co.za/2020/05/28/ban-on-tobacco-sales-inside-the-govt-s-court-papers
212	2020-05-27	Eye Witness News	Theto Mahlakoana	Scientific data, logic used for govt's tobacco sales ban decision - Dlamini-Zuma	https://ewn.co.za/2020/05/27/scientific-data-logic-used-for-govt-s-tobacco-sales-ban-decision-dlamini-zuma
213	2020-05-26	Eye Witness News	Theto Mahlakoana	BTFA: Govt consultations on cigarette sale ban a smokescreen	https://ewn.co.za/2020/05/26/btfa-govt-consultations-on-cigarette-sale-ban-a-smokescreen

214	2020-05-26	Eye Witness News	Bonga Dlulane	Steenhuisen: Reopening of economy about six weeks too late	https://ewn.co.za/2020/05/26/steenhuisen-reopening-of-economy-about-six-weeks-too-late
215	2020-05-26	Eye Witness News	Staff Reporter	OPINION: On COVID-19, science and the politics of data	https://ewn.co.za/2020/05/26/opinion-on-covid-19-science-and-the-politics-of-data
216	2020-05-26	Eye Witness News	Babalo Ndenze	Dlamini-Zuma defends cigarette sales ban, says she's not Mazzotti's friend	https://ewn.co.za/2020/05/26/dlamini-zuma-defends-cigarette-sales-ban-says-she-s-not-mazzotti-s-friend
217	2020-05-24	Eye Witness News	Sifiso Zulu	ANC 'dismayed' by attacks, defends Dlamini-Zuma in alcohol & tobacco sale debate	https://ewn.co.za/2020/05/24/anc-dismayed-by-attacks-defends-dlamini-zuma-in-alcohol-and-tobacco-sale-debate
218	2020-05-22	Eye Witness News	Lungelo Matangira	Tweeps see red over report Dlamini-Zuma wants booze, tobacco sold at level 1	https://ewn.co.za/2020/05/22/tweeps-see-red-over-report-dlamini-zuma-wants-booze-tobacco-sold-at-level-1
219	2020-05-22	Eye Witness News	Staff Reporter	230,000 charged for lockdown breaches, big drop in contact crimes, says Cele	https://ewn.co.za/2020/05/22/230-000-charged-for-lockdown-breaches-big-drop-in-contact-crimes-says-cele
220	2020-05-22	Eye Witness News	Judith February	JUDITH FEBRUARY: Mr President, the buck has to stop somewhere	https://ewn.co.za/2020/05/22/judith-february-mr-president-the-buck-has-to-stop-somewhere
221	2020-05-19	Eye Witness News	Adrian Bauman	RESEARCH CHECK: Does nicotine protect us against coronavirus?	https://ewn.co.za/2020/05/19/research-check-does-nicotine-protect-us-against-coronavirus
222	2020-05-16	Eye Witness News	Kaylynn Palm	Nearly 90% of SA smokers still getting cigarettes in lockdown – UCT survey	https://ewn.co.za/2020/05/16/nearly-90-of-sa-smokers-still-getting-cigarettes-in-lockdown-uct-survey
223	2020-05-15	Eye Witness News	Kaylynn Palm	It's lighters up as cigarette black market thrives under lockdown	https://ewn.co.za/2020/05/15/it-s-lighters-up-as-cigarette-black-market-thrives-under-lockdown

224	2020-05-13	Eye Witness News	Shamiela Fisher	WC police nab more suspects for cigarette smuggling	https://ewn.co.za/2020/05/13/wc-police-nab-more-suspects-for-cigarette-smuggling
225	2020-05-12	Eye Witness News	Theto Mahlakoana	Dlamini-Zuma ready to defend govt's decision on cigarette sales ban	https://ewn.co.za/2020/05/12/dlamini-zuma-ready-to-defend-govt-s-decision-on-cigarette-sales-ban
226	2020-05-12	Eye Witness News	Mia Lindeque	Govt to provide reasons for maintaining cigarette sales ban	https://ewn.co.za/2020/05/12/govt-to-provide-reasons-for-maintaining-cigarette-sales-ban
227	2020-05-07	Eye Witness News	Shamiela Fisher	Cigarette ban, court dates behind riot at EC's St Albans Prison	https://ewn.co.za/2020/05/07/cigarette-ban-court-dates-behind-riot-at-ec-s-st-albans-prison
228	2020-05-06	Eye Witness News	Keegan Leech	FACT CHECK: Can alcohol ban help South Africans fight COVID-19 infection?	https://ewn.co.za/2020/05/06/fact-check-can-alcohol-ban-help-south-africans-fight-covid-19-infection
229	2020-05-06	Eye Witness News	Staff Reporter	BATSA decides against legal action over cigarette sale ban	https://ewn.co.za/2020/05/06/batsa-decides-against-legal-action-over-cigarette-sale-ban
230	2020-06-21	Fin24	Murray Williams	Govt lawyers in sudden about-turn, get court to delay cigarette case until August	https://www.news24.com/fin24/companies/agribusiness/breaking-govt-lawyers-in-sudden-about-turn-get-court-to-delay-cigarette-case-until-august-20200626
231	2020-06-20	Fin24	Murray Williams	Illegal cigarette trade may reduce economic harm of tobacco ban - govt in court papers	https://www.news24.com/fin24/economy/illegal-cigarette-trade-may-reduce-economic-harm-of-tobacco-ban-govt-in-court-papers-20200620
232	2020-06-10	Fin24	Jan Cronje	Cigarette ban is both legal and supported by science, State argues in court	https://www.news24.com/fin24/economy/south-africa/cigarette-ban-is-both-legal-and-supported-by-science-state-argues-in-court-20200610

233	2020-06-08	Fin24	Jan Cronje	Tobacco group to argue Dlamini-Zuma overstepped powers in banning sales of cigarettes	https://www.news24.com/fin24/economy/tobacco-group-to-argue-dlamini-zuma-overstepped-powers-in-banning-cigarettes-20200608
234	2020-06-04	Fin24	Jan Cronje	Govt says lost tax revenue from smoking ban 'outweighed' by harm cigarettes cause	https://www.news24.com/fin24/economy/south-africa/govt-says-lost-tax-revenue-from-smoking-ban-outweighed-by-harm-cigarettes-cause-20200604
235	2020-05-16	Fin24	Marelise van der Merwe	Cigarette ban is 'failing', can create lasting illicit market - study	https://www.news24.com/fin24/Economy/South-Africa/cigarette-ban-is-failing-can-create-lasting-illicit-market-study-20200516
236	2020-05-04	Fin24	Lameez Omarjee	Ban on cigarette, liquor sales costing R1.5bn in lost tax revenue – Kieswetter	https://www.news24.com/fin24/economy/south-africa/ban-on-cigarette-and-liquor-sales-costing-r15-billion-in-lost-tax-revenue-kieswetter-20200430
237	2020-04-29	Fin24	Lameez Omarjee	Lockdown | Cigarettes, liquor still won't be for sale on Level 4	https://www.news24.com/fin24/Economy/South-Africa/lockdown-cigarettes-liquor-still-wont-be-for-sale-on-level-4-20200429
238	2020-04-26	Fin24	Nicole McCain	Lockdown: Smokers still able to get cigarettes, informal trade is active - survey	https://www.news24.com/news24/southafrica/news/lockdown-smokers-still-able-to-get-cigarettes-informal-trade-is-active-survey-20200426
239	2020-04-18	Fin24	Nicole McCain	Lockdown: Tobacco association to challenge cigarette ban in court	https://www.news24.com/news24/southafrica/news/lockdown-tobacco-association-to-challenge-cigarette-ban-in-court-20200418
240	2020-04-04	Fin24	Carin Smith	Cigarette ban could force 11 million smokers to seek illicit traders, industry warns	https://www.news24.com/fin24/companies/agribusiness/cigarette-ban-could-force-11-million-smokers-to-seek-illicit-traders-industry-warns-20200404

241	2020-06-10	Fin24	Ernest Mabuza	Judgment reserved: the battle of wills in court over tobacco ban	https://www.sowetanlive.co.za/news/south-africa/2020-06-10-judgment-reserved-the-battle-of-wills-in-court-over-tobacco-ban/
242	2020-05-04	Fin24	Lameez Omarjee	If cigarette sales spread coronavirus, prove it – tobacco association	https://www.news24.com/fin24/economy/south-africa/if-cigarette-sales-spread-coronavirus-prove-it-tobacco-association-20200504
243	2020-05-03	Fin24	Jenni Evans	ANCWL fuming over treatment of Dlamini-Zuma on cigarette about-turn	https://www.news24.com/news24/southafrica/news/ancwl-fuming-over-treatment-of-dlamini-zuma-on-cigarette-about-turn-20200503
244	2020-05-11	Fin24	Jan Cronje	Nothing sinister' behind renewed cigarette ban, says Dlamini-Zuma ahead of court case	https://www.news24.com/fin24/economy/nothing-sinister-behind-renewed-cigarette-ban-says-dlamini-zuma-ahead-of-court-case-20200511
245	2020-05-23	Fin24	Canny Maphanga	Lockdown: Informal Traders Alliance pleads with NCCC to lift tobacco ban	https://www.news24.com/news24/southafrica/news/lockdown-informal-traders-alliance-pleads-with-nccc-to-lift-tobacco-ban-20200523
246	2020-05-24	Fin24	Staff Reporter	Lockdown: Draft regulations propose tobacco ban extension, but lifting ban on liquor - report	https://www.news24.com/news24/southafrica/news/lockdown-draft-regulations-propose-tobacco-ban-extension-but-lifting-ban-on-liquor-report-20200524
247	2020-08-18	Fin24	Alex Mitchley	Smokers stock up out of fear the government will reverse unbanning order	https://www.news24.com/news24/southafrica/news/smokers-stock-up-out-of-fear-the-government-will-reverse-unbanning-order-20200818
248	2020-08-17	Fin24	Mandisa Nyathi	It's official: Level 2's booze, smokes and local travel have been gazetted	https://www.news24.com/citypress/news/its-official-level-2s-booze-smokes-and-local-travel-have-been-gazetted-20200817

249	2020-09-08	Fin24	Staff Reporter	Coronavirus morning update: Bans created new criminal networks, and global economies take strain	https://www.news24.com/health24/medical/infectious-diseases/coronavirus/coronavirus-morning-update-bans-created-new-criminal-networks-and-global-economies-take-strain-20200908-5
250	2020-08-15	Fin24	Kaveel Singh	SA down to Level 2 lockdown, with tobacco, alcohol bans lifted 15 Aug	https://www.news24.com/news24/southafrica/news/sa-down-to-level-2-lockdown-with-tobacco-alcohol-bans-lifted-20200815
251	2020-12-11	Fin24	Londiwe Buthelez	Tobacco sales ban was unconstitutional and unnecessary, court finds	https://www.news24.com/fin24/economy/tobacco-ban-was-unconstitutional-and-uneccessary-says-court-20201211
252	2020-08-05	Fin24	Anathi Madubela	Ackerman lashes out at 'confusing, contradictory' explanations for tobacco, alcohol sales ban | Fin24	https://www.news24.com/fin24/companies/retail/ackerman-lashes-out-at-confusing-contradictory-explanations-for-tobacco-alcohol-sales-ban-20200805
253	2020-06-25	Fin24	Siphesihle Ndamase	Scientist says there are more merits in banning alcohol than tobacco	https://briefly.co.za/69283-scientist-merits-banning-alcohol-tobacco.html
254	2020-05-07	Fin24	Riaan Grobler	eNCA morning anchors off air but 'not suspended', following Nkosazana Dlamini-Zuma remarks	https://www.news24.com/news24/SouthAfrica/News/enca-morning-anchors-off-air-but-not-suspended-following-nkosazana-dlamini-zuma-remarks-20200507
255	2020-08-16	Fin24	Sibongile Khumalo and Lameez Omarjee	Thanks for reopening the economy, but we need more public investment - Cosatu	https://www.news24.com/fin24/economy/thanks-for-reopening-the-economy-but-we-need-more-public-investment-cosatu-20200816
256	2020-04-30	Health24	Jan Gerber	Tobacco ban: 'We understood the reasons and we agree,' says parliamentary health committee chair	https://www.news24.com/news24/southafrica/news/tobacco-ban-we-understood-the-reasons-and-we-agree-says-parliamentary-health-committee-chair-20200430
257	2020-05-01	Health24	Jan Gerber	President Ramaphosa supports tobacco ban - Jackson Mthembu	https://www.news24.com/news24/southafrica/news/president-ramaphosa-supports-tobacco-ban-jackson-mthembu-20200501

258	2020-06-18	Health24	Jan Gerber	Covid-19: No information was withheld from public, Ramaphosa tells Parliament | News24	https://www.news24.com/news24/southafrica/news/covid-19-no-information-was-withheld-from-public-ramaphosa-tells-parliament-20200618
259	2020-04-21	Independent Online	Jonisayi Maromo	Call to lift ban on cigarettes, non-essential goods as SA waits for Ramaphosa's speech	https://www.iol.co.za/news/politics/call-to-lift-ban-on-cigarettes-non-essential-goods-as-sa-waits-for-ramaphosas-speech-46993082
260	2020-06-28	Independent Online	Bongani Hans	Fight on tobacco ban turns into blame game after judgment	https://www.iol.co.za/news/politics/fight-on-tobacco-ban-turns-into-blame-game-after-judgment-50051139
261	2020-05-28	Independent Online	Samkelo Mtshali	Malema praises Dlamini Zuma for sticking to her guns on tobacco ban	https://www.iol.co.za/news/politics/malema-praises-dlamini-zuma-for-sticking-to-her-guns-on-tobacco-ban-48633902
262	2020-07-01	Independent Online	Kelly-Jane Turner	Cigarette ban will eventually be lifted, says Ramaphosa	https://www.iol.co.za/news/politics/cigarette-ban-will-eventually-be-lifted-says-ramaphosa-50248393
263	2020-05-04	Independent Online	Sihle Mavuso	Fire rages over tobacco ban during lockdown	https://www.iol.co.za/news/politics/fire-rages-over-tobacco-ban-during-lockdown-47555402
264	2020-04-30	Independent Online	Samkelo Mtshali	Praise for Dlamini Zuma's stance on continued tobacco ban	https://www.iol.co.za/news/politics/praise-for-dlamini-zumas-stance-on-continued-tobacco-ban-47410646
265	2020-05-05	Independent Online	Marvin Charles	Ill-considered tobacco ban impacts health, welfare of millions across SA	https://www.iol.co.za/capeargus/news/ill-considered-tobacco-ban-impacts-health-welfare-of-millions-across-sa-47558570
266	2020-05-30	Independent Online	Zelda Venter	Tobacco sales debate set for court	https://www.iol.co.za/pretoria-news/news/tobacco-sales-debate-set-for-court-48712225

267	2020-07-05	Independent Online	African News Agency	#TheBanMustFall open letter to President Ramaphosa details devastating impact of tobacco ban	https://www.iol.co.za/news/politics/thebanmustfall-open-letter-to-president-ramaphosa-details-devastating-impact-of-tobacco-ban-50460645
268	2020-06-11	Independent Online	Baldwin Ndaba	Tobacco ban was to save lives in the country, court told	https://www.iol.co.za/news/politics/tobacco-ban-was-to-save-lives-in-the-country-court-told-49249981
269	2020-06-03	Independent Online	Lyse Comins And ANA	Court to hear challenge to cigarette ban	https://www.iol.co.za/mercury/news/court-to-hear-challenge-to-cigarette-ban-48909322
270	2020-08-05	Independent Online	ANA Reporter	BATSA rubbishes Dlamini Zuma's 'perverse justification' for tobacco ban in court	https://www.iol.co.za/news/south-africa/batsa-rubbishes-dlamini-zumas-perverse-justification-for-tobacco-ban-in-court-2fbad1be-0b60-4ba9-855b-972cc2d1fa47
271	2020-05-27	Independent Online	Staff Reporter	AfriForum serves lawyer's letter on Dlamini Zuma, threatens legal action if tobacco ban is not lifted	https://www.iol.co.za/news/south-africa/afriforum-serves-lawyers-letter-on-dlamini-zuma-threatens-legal-action-if-tobacco-ban-is-not-lifted-48586318
272	2020-07-24	Independent Online	Helene Eloff	Tobacco ban is missed opportunity to create smoke-free SA - PMSA	https://www.iol.co.za/technology/gadgets/tobacco-ban-is-missed-opportunity-to-create-smoke-free-sa-pmsa-1a53be71-aaad-4e2a-8bbe-ef7bef051f61
273	2020-06-09	Independent Online	Zintle Mahlati	High Court to hear Fita case on tobacco ban on Wednesday	https://www.iol.co.za/news/politics/high-court-to-hear-fita-case-on-tobacco-ban-on-wednesday-49153036
274	2020-07-03	Independent Online	Zintle Mahlati	Fita has filed appeal over cigarette sales ban ruling	https://www.iol.co.za/news/politics/fita-has-filed-appeal-over-cigarette-sales-ban-ruling-50356392

275	2020-06-10	Independent Online	Sandile Mchunu	British American Tobacco puts pressure on share with cut in its revenue expectations	https://www.iol.co.za/business-report/companies/british-american-tobacco-puts-pressure-on-share-with-cut-in-its-revenue-expectations-49175982
276	2020-07-21	Independent Online	African News Agency	UCT study stubs out argument for continued cigarette ban	https://www.iol.co.za/news/politics/uct-study-stubs-out-argument-for-continued-cigarette-ban-c5619f0d-4b73-4c2c-a860-93223b2323e1
277	2020-07-23	Independent Online	ANA Reporter	Tobacco ban costing South Africa R35m a day in lost taxes - BATSA	https://www.iol.co.za/news/politics/tobacco-ban-costing-south-africa-r35m-a-day-in-lost-taxes-batsa-ff5d9355-1364-4f5e-abb0-18941d98d031
278	2020-09-29	Independent Online	ANA Reporter	FITA terminates Gold Leaf's membership as rumours swirl about illegal sales during tobacco ban	https://www.iol.co.za/news/south-africa/western-cape/fita-terminates-gold-leafs-membership-as-rumours-swirl-about-illegal-sales-during-tobacco-ban-ec14a361-f1be-5879-9f4d-98d9f4abead7
279	2020-04-07	Independent Online	Zelda Venter	Coronavirus: No booze, smokes or walkies, now a ban on kisses?	https://www.iol.co.za/pretoria-news/coronavirus-no-booze-smokes-or-walkies-now-a-ban-on-kisses-46426133
280	2020-04-04	Independent Online	SAPeople Editorial	Calls for Unbanning of 'Non-Essential' Goods Like Cigarettes in Retail Stores Currently Trading	https://www.sapeople.com/2020/04/04/calls-for-unbanning-of-non-essential-goods-like-cigarettes-in-retail-stores-currently-trading/
281	2020-05-28	Independent online	Bradley Slade	Court may struggle to find strong enough link between ban on tobacco and its intended purpose	https://www.iol.co.za/news/politics/opinion/court-may-struggle-to-find-strong-enough-link-between-ban-on-tobacco-and-its-intended-purpose-48589915
282	2020-05-02	Independent online	Sameer Naik and Tanya Waterworth	Tobacco big guns on fire over cigarette ban	https://www.iol.co.za/ios/news/tobacco-big-guns-on-fire-over-cigarette-ban-47468425

283	2020-07-07	Independent Online	Staff Reporter	Govt Promises To Consider Input As It Seeks Comment On Lockdown Regulations	https://iafrica.com/govt-promises-to-consider-input-as-it-seeks-comment-on-lockdown-regulations/
284	2020-05-11	Independent Online	Nomahlubi Jordaan	Ban on cigarettes a crucial part of fighting Covid-19: HSRC	https://www.heraldlive.co.za/news/2020-05-11-ban-on-cigarettes-a-crucial-part-of-fighting-covid-19-hsrc/
285	2020-05-06	Independent Online	Naledi Shange	British American Tobacco pauses court battle over cigarette ban	https://www.heraldlive.co.za/news/2020-05-06-british-american-tobacco-pauses-court-battle-over-cigarette-ban/
286	2020-05-01	Independent Online	Thabo Mokone And Naziziphiwo Buso	Mboweni does not support alcohol, cigarette sales ban	https://www.heraldlive.co.za/weekend-post/your-weekend/2020-05-01-mboweni-does-not-support-alcohol-cigarette-sales-ban/
287	2020-04-30	Independent Online	Thabo Mokone	Tito Mboweni reveals he does not support ban on alcohol and cigarette sales	https://www.heraldlive.co.za/news/politics/2020-04-30-tito-mboweni-reveals-he-does-not-support-ban-on-alcohol-and-cigarette-sales/
288	2020-04-30	Independent Online	Nomahlubi Jordaan	Tobacco association heads to court to challenge ban on cigarette sales	https://www.heraldlive.co.za/news/2020-04-30-tobacco-association-heads-to-court-to-challenge-ban-on-cigarette-sales/
289	2020-04-30	Independent Online	Nomahlubi Jordaan	More than 270,000 South Africans sign petition against ban on cigarette sales	https://www.heraldlive.co.za/news/2020-04-30-over-270000-south-africans-sign-petition-against-ban-on-cigarette-sales/
290	2020-04-24	Independent Online	Staff Reporter	Covid-19 | Cigarette industry drops legal challenge as smokes set to return to shelves	https://www.heraldlive.co.za/news/2020-04-24-covid-19-cigarette-industry-drops-legal-challenge-as-smokes-set-to-return-to-shelves/
291	2020-04-20	Independent Online	Cebelihle Bhengu	POLL | Should the lockdown ciggie ban be lifted?	https://www.heraldlive.co.za/news/2020-04-20-poll-should-the-lockdown-ciggie-ban-be-lifted/

292	2020-06-26	Independent Online	ANA Reporter	Cigarette industry in shock after double court blow for overturning smoking ban	https://www.iol.co.za/news/politics/cigarette-industry-in-shock-after-double-court-blow-for-overturning-smoking-ban-49990370
293	2020-05-30	Independent Online	Ziyanda Mgandela	Recovering drug addict's smoking plea to president	https://www.iol.co.za/ios/news/recovering-drug-addicts-smoking-plea-to-president-48709080
294	2020-08-19	Independent Online	Staff Reporter	SA smokers empty shops of cigarettes after ban lifted	https://www.iol.co.za/business-report/companies/sa-smokers-empty-shops-of-cigarettes-after-ban-lifted-6b696acb-187e-4583-9a9e-69f66a8b1e37
295	2020-04-29	Independent Online	Lou-Anne Daniels	Ban on alcohol and cigarette sales remains in place under Level 4 lockdown	https://www.iol.co.za/news/politics/ban-on-alcohol-and-cigarette-sales-remains-in-place-under-level-4-lockdown-47362327
296	2020-05-04	Independent Online	Sihle Mavuso	Ramaphosa backs Dlamini-Zuma in cigarette ban war	https://www.iol.co.za/news/politics/ramaphosa-backs-dlamini-zuma-in-cigarette-ban-war-47507125
297	2020-05-30	Independent Online	Staff Reporter	Tobacco ban battle set for court	https://www.iol.co.za/ios/news/tobacco-ban-battle-set-for-court-48709054
298	2020-10-29	Independent Online	Zacharia Motsumi	SA needs new post Covid-19 approach to growing illicit tobacco sector	https://www.iol.co.za/the-star/opinion-analysis/sa-needs-new-post-covid-19-approach-to-growing-illicit-tobacco-sector-6cc4b095-486f-42cf-a619-015e2e21e30a
299	2020-05-26	Independent Online	Zintle Mahlati	Dlamini Zuma insists on tobacco ban, denies friendship with Mazzotti	https://www.iol.co.za/news/politics/dlamini-zuma-insists-on-tobacco-ban-denies-friendship-with-mazzotti-48520842
300	2020-06-25	Independent Online	IOL Reporter	New cigarette ban petition takes aim at judges in Fita case	https://www.iol.co.za/news/politics/new-cigarette-ban-petition-takes-aim-at-judges-in-fita-case-49921779
301	2020-08-06	Independent Online	Brandstories	Cigarette producers call for end to lockdown ban	https://www.iol.co.za/news/partnered/cigarette-producers-call-for-end-to-lockdown-ban--eacf0ee4-b3e8-4395-b2fa-bcde063e04be

302	2020-05-06	Independent Online	Sihle Mavuso	BAT no longer going to court over cigarette sale ban	https://www.iol.co.za/news/politics/bat-no-longer-going-to-court-over-cigarette-sale-ban-47605186
303	2020-06-08	Independent Online	Baldwin Ndaba	Fita to oppose attempts to postpone court hearing about lifting cigarette sales ban	https://www.iol.co.za/news/politics/fita-to-oppose-attempts-to-postpone-court-hearing-about-lifting-cigarette-sales-ban-49135685
304	2020-07-22	Independent Online	Zintle Mahlati	Government backtracks on tweet about alcohol, cigarette ban	https://www.iol.co.za/news/politics/government-backtracks-on-tweet-about-alcohol-cigarette-ban--e7b44c33-a7a2-4837-9e98-9da5222cce5a
305	2020-04-29	Independent Online	Marvin Charles	SA loses R36m a day due to the sale of illicit cigarettes amid lockdown	https://www.iol.co.za/capeargus/news/sa-loses-r36m-a-day-due-to-the-sale-of-illicit-cigarettes-amid-lockdown-47379414
306	2020-07-15	Independent Online	Zelda Venter	Fita states its case for leave to appeal ban on cigarettes	https://www.iol.co.za/pretoria-news/news/fita-states-its-case-for-leave-to-appeal-ban-on-cigarettes-51006347
307	2020-05-01	Independent Online	Lou-Anne Daniels	Lockdown level 4: BAT threatens court action over ban on cigarette sales	https://www.iol.co.za/news/politics/lockdown-level-4-bat-threatens-court-action-over-ban-on-cigarette-sales-47456509
308	2020-06-04	Independent Online	Loyiso Sidimba	Cigarette sale ban: BATSA launches another attack	https://www.iol.co.za/news/politics/cigarette-sale-ban-batsa-launches-another-attack-48964359
309	2020-07-24	Independent Online	Lou-Anne Daniels	'Not surprised': FITA heads to Supreme Court of Appeal over ban on cigarette sales	https://www.iol.co.za/news/politics/not-surprised-fita-heads-to-supreme-court-of-appeal-over-ban-on-cigarette-sales-ad446ef3-c575-493f-a956-8d7cabf147d3
310	2020-05-27	Independent Online	ANA Reporter	Agricultural union requests judicial commission on illegal trade in cigarettes	https://www.iol.co.za/news/politics/agricultural-union-requests-judicial-commission-on-illegal-trade-in-cigarettes-48568450

311	2020-06-01	Independent Online	ANA Reporter	BATSA wants court case on banning of cigarettes heard on June 22	https://www.iol.co.za/news/south-africa/batsa-wants-court-case-on-banning-of-cigarettes-heard-on-june-22-48826334
312	2020-05-12	Independent Online	Baldwin Ndaba	Legal showdown between government and Fita put on hold for now	https://www.iol.co.za/news/politics/legal-showdown-between-government-and-fita-put-on-hold-for-now-47839831
313	2020-05-22	Independent Online	Staff Reporter	Cigarette and alcohol sales may remain banned until 2021 - FMF CEO	https://www.iol.co.za/news/south-africa/kwazulu-natal/cigarette-and-alcohol-sales-may-remain-banned-until-2021-fmf-ceo-48404829
314	2020-04-30	Independent Online	IOL reporter	Mboweni 'lost debate' on allowing sale of alcohol, cigarettes under Level 4	https://www.iol.co.za/news/politics/mboweni-lost-debate-on-allowing-sale-of-alcohol-cigarettes-under-level-4-47406461
315	2020-04-18	Independent Online	Sameer Naik	We are going to court,' says Tobacco Association over cigarette ban during lockdown	https://www.iol.co.za/saturday-star/news/we-are-going-to-court-says-tobacco-association-over-cigarette-ban-during-lockdown-46869185
316	2020-03-31	Independent Online	Staff Reporter	Plea to repeal ban on sale of alcohol, cigarettes due to 'risks, discrimination'	https://www.iol.co.za/capetimes/news/plea-to-repeal-ban-on-sale-of-alcohol-cigarettes-due-to-risks-discrimination-45813186
317	2020-07-08	Independent Online	Staff Reporter	Soapie actor among 5 nabbed for dealing in illicit cigarettes at Limpopo taxi rank	https://www.iol.co.za/news/south-africa/limpopo/soapie-actor-among-5-nabbed-for-dealing-in-illicit-cigarettes-at-limpopo-taxi-rank-50593632
318	2020-08-07	Independent Online	Marvin Charles	BATSA: 'What level of justification do you need for this tobacco ban?'	https://www.iol.co.za/capeargus/news/batsa-what-level-of-justification-do-you-need-for-this-tobacco-ban-4998fe14-0606-4851-8558-08f53a5571ad

319	2020-08-26	Independent Online	Zelda Venter	Fita withdraws pending appeal on sale of tobacco after settlement	https://www.iol.co.za/pretoria-news/news/fita-withdraws-pending-appeal-on-sale-of-tobacco-after-settlement-6ab48e2d-4fa1-453d-87c5-afdc8c7781ee
320	2020-10-07	Independent Online	IOL	Tobacco companies in SA may have aided, abetted illicit cigarette sales, report says	https://www.iol.co.za/news/south-africa/tobacco-companies-in-sa-may-have-aided-abetted-illicit-cigarette-sales-report-says-07fd89c8-6ab5-47fd-a21e-a189e69772e2
321	2020-05-25	Independent Online	Baldwin Ndaba	Tobacco industry guns for Ramaphosa in court	https://www.iol.co.za/news/politics/tobacco-industry-guns-for-ramaphosa-in-court-48507329
322	2020-08-05	Independent Online	Baldwin Ndaba	Pick n Pay boss enters liquor, cigarettes fray	https://www.iol.co.za/business-report/companies/pick-n-pay-boss-enters-liquor-cigarettes-fray-d871551a-281c-4828-8e12-6704cfd5b2ca
323	2020-08-07	Independent Online	ANA Reporter	Dlamini Zuma submits affidavit to Appeal Court, BATSA hoping for 'swift end' to tobacco sales ban	https://www.iol.co.za/news/south-africa/dlamini-zuma-submits-affidavit-to-appeal-court-batsa-hoping-for-swift-end-to-tobacco-sales-ban-022575d0-86d6-4f49-93f1-a319b6547de9
324	2020-05-12	Independent Online	Baldwin Ndaba	Fita seeks minutes of meeting where decision was taken to continue cigarette sale ban	https://www.iol.co.za/news/politics/fita-seeks-minutes-of-meeting-where-decision-was-taken-to-continue-cigarette-sale-ban-47876693
325	2020-07-09	Independent Online	ANA Reporter	Gauteng High Court, Pretoria to hear tobacco ban appeal	https://www.iol.co.za/pretoria-news/gauteng-high-court-pretoria-to-hear-tobacco-ban-appeal-50741497
326	2020-05-05	Independent Online	Sihle Mavuso	FITA says tobacco ban is increasing illicit trade of cigarettes	https://www.iol.co.za/news/politics/fita-says-tobacco-ban-is-increasing-illicit-trade-of-cigarettes-47585340

327	2020-05-11	Independent Online	Edward West	Smoking ban proves to be efficient, study finds	https://www.iol.co.za/business-report/economy/smoking-ban-proves-to-be-efficient-study-finds-47814349
328	2020-05-24	Independent Online	ANA Reporter	From which planet does our lockdown tobacco law come from? asks FMF	https://www.iol.co.za/news/south-africa/from-which-planet-does-our-lockdown-tobacco-law-come-from-asks-fmf-48456759
329	2020-07-04	Independent Online	Sameer Naik	Fita determined to go ahead with fight against tobacco ban	https://www.iol.co.za/saturday-star/news/fita-determined-to-go-ahead-with-fight-against-tobacco-ban-50387160
330	2020-08-04	Independent Online	ANA Reporter	Pick n Pay retrenches staff and implores government to review tobacco, alcohol bans	https://www.iol.co.za/business-report/companies/pick-n-pay-retrenches-staff-and-implores-government-to-review-tobacco-alcohol-bans-4772ecfa-2d97-44c8-a729-1086580a96c4
331	2020-05-05	Independent Online	Professor Francois Steyn And Harriét Klopper	Cigarette ban backfiring with 7 million smokers against government decision	https://www.iol.co.za/capeargus/opinion/cigarette-ban-backfiring-with-7-million-smokers-against-government-decision-47545171
332	2020-07-06	Independent Online	Lou-Anne Daniels	As tobacco ban drags on, these smokers are demanding to have their voice heard	https://www.iol.co.za/news/politics/as-tobacco-ban-drags-on-these-smokers-are-demanding-to-have-their-voice-heard-50495363
333	2020-07-06	Independent Online	Lou-Anne Daniels	As tobacco ban drags on, these smokers are demanding to have their voice heard	https://www.iol.co.za/news/politics/as-tobacco-ban-drags-on-these-smokers-are-demanding-to-have-their-voice-heard-50495363
334	2020-07-05	Independent Online	Reeshnie Chetty	Smoking allowed at Durban casinos despite sale ban	https://www.iol.co.za/sunday-tribune/news/smoking-allowed-at-durban-casinos-despite-sale-ban-50449274

335	2020-05-26	Independent Online	Marvin Charles	Vapour products industry tears into continued level 3 ban	https://www.iol.co.za/capeargus/news/vapour-products-industry-tears-into-continued-level-3-ban-48509313
336	2020-08-12	Independent Online	Staff Reporter	Tobacco products worth over R1m seized near Paarl, four held in separate incidents	https://www.iol.co.za/news/south-africa/western-cape/tobacco-products-worth-over-r1m-seized-near-paarl-four-held-in-separate-incidents-37acd6c8-670d-4646-a399-d41165977f68
337	2020-06-18	Independent Online	Staff Reporter	R1.3m contraband cigarettes confiscated at Farazella border	https://www.iol.co.za/news/south-africa/r13m-contraband-cigarettes-confiscated-at-farazella-border-49586229
338	2020-05-03	Independent Online	Manyane Manyane	Lawyers threaten Ramaphosa's National Command Council	https://www.iol.co.za/sundayindependent/news/lawyers-threaten-ramaphosas-national-command-council-47489065
339	2020-01-05	Independent Online	Marvin Charles	Ramaphosa, Dlamini-Zuma head to SCA over tobacco ban ruling	https://www.iol.co.za/news/politics/ramaphosa-dlamini-zuma-head-to-sca-over-tobacco-ban-ruling-322316c5-4d67-49fb-94ff-0f511c82535d
340	2020-08-20	Mail & Guardian	Tshegofatso Mathe	Sars plan for illicit tobacco still being refined	https://mg.co.za/business/2020-08-22-sars-plan-for-illicit-tobacco-still-being-refined/
341	2020-08-07	Mail & Guardian	Special Reports	Smokers hold thumbs for cigarette ban to be lifted	https://mg.co.za/special-reports/2020-08-07-smokers-hold-thumbs-for-cigarette-ban-to-be-lifted/
342	2020-06-05	Mail & Guardian	Tshegofatso Mathe	Cigarette sales ban battle looms	https://mg.co.za/business/2020-06-05-cigarette-sales-ban-battle-looms/
343	2020-05-28	Mail & Guardian	Alex Norcia	How South Africa’s Coronavirus Tobacco Prohibition Backfired	https://filtermag.org/south-africa-coronavirus-cigarettes-ban/
344	2020-05-01	News24	Jan Gerber	Bizarre and irregular' - British American Tobacco on government's tobacco ban	https://www.news24.com/news24/southafrica/news/bizarre-and-irregular-british-american-tobacco-on-governments-tobacco-ban-20200501

345	2020-05-04	News24	Staff Reporter	Second tobacco company takes on government on urgent basis	https://www.news24.com/citypress/news/second-tobacco-company-takes-on-government-on-urgent-basis-20200504
346	2020-05-04	News24	Staff Reporter	Tobacco ban was not taken lightly – Ramaphosa	https://www.news24.com/citypress/news/tobacco-ban-was-not-taken-lightly-ramaphosa-20200504
347	2020-05-02	News24	Staff Reporter	Coronavirus morning update: Level 4 lockdown just started but already a warning - reversing it is 'easy'	https://www.health24.com/Medical/Infectious-diseases/Coronavirus/coronavirus-morning-update-level-4-lockdown-just-started-but-already-a-warning-reversing-it-is-easy-20200502-2
348	2020-05-06	News24	Jan Cronje	SA's largest cigarette company drops proposed legal action against tobacco ban	https://www.news24.com/fin24/economy/sas-largest-cigarette-company-drops-proposed-legal-action-against-tobacco-ban-20200506
349	2020-05-06	News24	Phakamani Mvelashe	Free State man on buying cigarettes from the black market: “I can’t quit”	https://www.news24.com/drum/news/free-state-man-on-buying-cigarettes-from-the-black-market-i-cant-quit-20200506
350	2020-05-08	News24	Lizeka Tandwa	Ramaphosa hit with another letter questioning lawfulness of the NCCC	https://www.news24.com/news24/southafrica/news/ramaphosa-hit-with-another-letter-questioning-lawfulness-of-the-nccc-20200508
351	2020-05-10	News24	Alex Mitchley and Murray Williams	Smoke and mirrors: 4 minutes – that's how long it took to find banned cigarettes during lockdown	https://www.news24.com/news24/southafrica/news/smoke-and-mirrors-4-minutes-thats-how-long-it-took-to-find-banned-cigarettes-during-lockdown-20200510
352	2020-08-17	News24	Khulekani Magubane	Tensions loom between tobacco producers as ban lifts	https://www.news24.com/fin24/companies/industrial/tensions-loom-between-tobacco-producers-and-ban-lifts-20200817

353	2020-07-09	News24	Jan Cronje	Court agrees to hear bid by tobacco group for leave to appeal ruling that kept cigarettes banned	https://www.news24.com/fin24/economy/court-agrees-to-hear-bid-by-tobacco-group-for-leave-to-appeal-ruling-that-kept-cigarettes-banned-20200709
354	2020-06-26	News24	Londiwe Buthelezi	Government denies delaying BATSA tobacco ban case	https://www.news24.com/fin24/economy/government-denies-delaying-batsa-tobacco-ban-case-20200626
355	2020-06-25	News24	Murray Williams & Marelise van der Merwe	BATSA, Dlamini-Zuma set to face off in court next week over cigarette ban	https://www.news24.com/fin24/companies/agribusiness/just-in-batsa-dlamini-zuma-set-to-face-off-in-court-next-week-20200625
356	2020-09-07	News24	Prinesha Naidoo, Bloomberg	SA booze, tobacco ban created new criminal networks	https://www.news24.com/fin24/economy/sa-booze-tobacco-ban-created-new-criminal-networks-20200907
357	2020-09-03	News24	Khulekani Magubane	It's not just the economy, Kieswetter says on SARS collection troubles	https://www.news24.com/fin24/economy/south-africa/its-not-just-the-economy-kieswetter-says-on-sars-collection-troubles-20200903
358	2020-08-19	News24	Khulekani Magubane	Illicit smokes will not vanish overnight, warns BATSA boss as ban lifted	https://www.news24.com/fin24/economy/south-africa/illicit-smokes-will-not-vanish-overnight-warns-batsa-boss-as-ban-lifted-20200819
359	2020-08-17	News24	Staff Reporter	All the new rules for Level 2, including how many people can come over for a braai	https://www.businessinsider.co.za/level-2-lockdown-rules-2020-8
360	2020-08-16	News24	Azarrah Karrim	Lockdown: Level 2 officially starts on Tuesday morning	https://www.news24.com/news24/southafrica/news/lockdown-level-2-officially-starts-on-tuesday-morning-20200816
361	2020-08-16	News24	Palesa Dlamini	Move to level 2 nothing to be applauded, say opposition parties	https://www.news24.com/citypress/news/move-to-level-2-nothing-to-be-applauded-say-opposition-parties-20200816

362	2020-08-16	News24	Jan Gerber	Too little, too late - opposition responds to easing of lockdown regulations	https://www.news24.com/news24/southafrica/news/too-little-too-late-opposition-responds-to-easing-of-lockdown-regulations-20200816
363	2020-05-18	News24	Mandisa Nyathi	Surge in patients being treated for illnesses related to home-made booze	https://www.news24.com/citypress/news/surge-in-patients-being-treated-for-illnesses-related-to-home-made-booze-20200518
364	2020-08-05	News24	Queenin Masuabi	Tobacco industry given confidence boost by chance to appeal ban	https://www.news24.com/citypress/news/tobacco-industry-given-confidence-boost-by-chance-to-appeal-ban-20200805
365	2020-07-17	News24	Azarrah Karrim	NICD director cautions against smoking, says getting evidence of links to 'severe' Covid-19 is hard	https://www.news24.com/news24/southafrica/news/no-direct-evidence-linking-smoking-with-severe-covid-19-but-not-a-good-idea-says-nicd-director-20200717
366	2020-07-13	News24	Penelope Mashego	We were blindsided by fresh ban, says alcohol industry	https://www.news24.com/fin24/companies/agribusiness/we-were-blindsided-by-fresh-ban-says-alcohol-industry-20200713
367	2020-06-23	News24	Jan Gerber	Dodging questions: Dlamini-Zuma does not have available the reasons a state of disaster was declared	https://www.news24.com/news24/southafrica/news/dodging-questions-dlamini-zuma-does-not-have-the-reasons-a-state-of-disaster-was-declared-available-20200623
368	2020-06-11	News24	Londiwe Buthelezi	ANALYSIS | Here's how the state justified the tobacco ban in court. Will it be enough?	https://www.news24.com/fin24/economy/analysis-heres-how-the-state-justified-the-tobacco-ban-in-court-will-it-be-enough-20200611
369	2020-06-08	News24	Gcina Ntsaluba	Tobacco ban creates flourishing illicit market	https://www.news24.com/citypress/news/tobacco-ban-creates-flourishing-illicit-market-20200608

370	2020-06-04	News24	Azarrah Karrim	Lockdown: AfriForum lays charges with UN human rights body over 'draconic regulations'	https://www.news24.com/news24/southafrica/news/lockdown-afriforum-lays-charges-with-un-human-rights-body-over-draconic-regulations-20200604
371	2020-05-29	News24	Lizeka Tandwa	Cigarette sales ban: DA labels Nkosazana Dlamini-Zuma a 'liar', says Ramaphosa should 'fire' her	https://www.news24.com/news24/southafrica/news/cigarette-sales-ban-da-labels-nkosazana-dlamini-zuma-a-liar-says-ramaphosa-should-fire-her-20200529
372	2020-05-11	News24	Lameez Omarjee and Jan Cronje	Lockdown | Govt agrees to provide reasons for ban on cigarette sales	https://www.news24.com/fin24/Economy/lockdown-govt-agrees-to-provide-reasons-for-ban-on-cigarette-sales-20200511
373	2020-08-02	News24	Murray Williams	Lockdown cigarette wars: Govt admits smokers 'less likely to be infected' with coronavirus | News24	https://www.news24.com/news24/southafrica/news/lockdown-cigarette-wars-govt-admits-smokers-less-likely-to-be-infected-with-coronavirus-20200802
374	2020-07-02	News24	Riaan Grobler	Lockdown: Public concern about rise in violence following lifting of booze ban, survey finds	https://www.news24.com/news24/southafrica/news/lockdown-public-concern-about-rise-in-violence-following-lifting-of-booze-ban-survey-finds-20200702
375	2020-05-24	News24	Lameez Omarjee	Lockdown | Cigarette ban to remain under level 3, but you can buy booze	https://www.news24.com/fin24/Economy/lockdown-cigarette-ban-to-remain-under-level-3-but-you-can-buy-booze-20200524
376	2020-08-19	Reuters	Tumelo Modiba and Promit Mukherjee	South African smokers empty shops of cigarettes after ban lifted - Reuters Africa	https://www.reuters.com/article/us-health-coronavirus-safrica-tobacco-idUSKCN25E2FM
377	2020-08-06	SABC News	SABC News	Cigarette sales ban legal battle continues on Thursday	https://www.sabcnews.com/sabcnews/cigarette-sales-ban-legal-battle-continues-on-thursday/
378	2020-05-05	SABC News	Naledi Ngcobo	Tobacco lobby groups gear up to fight cigarette sale ban	https://www.sabcnews.com/sabcnews/tobacco-lobby-groups-gear-up-to-fight-cigarette-sale-ban/

379	2020-05-02	SABC News	Staff Reporter	British American Tobacco threatens legal action over cigarette ban	https://www.sabcnews.com/sabcnews/british-american-tobacco-threatens-legal-action-over-cigarette-ban/
380	2020-10-30	SABC News	Tashlan Naidoo	Lobby group calls for 100% tax hike on tobacco products	https://www.sabcnews.com/sabcnews/lobby-group-calls-for-100-tax-hike-on-tobacco-products/
381	2020-08-18	SABC News	Staff Reporter	Dlamini-Zuma defends five month ban on sale of cigarettes	https://www.sabcnews.com/sabcnews/dlamini-zuma-defends-five-month-ban-on-sale-of-cigarettes/
382	2020-08-17	SABC News	Staff Reporter	Tobacco industry relieved ban has been lifted	https://www.sabcnews.com/sabcnews/tobacco-industry-relieved-ban-has-been-lifted/
383	2020-08-15	SABC News	Aphumelele Mdlalane	SCA grants FITA leave to appeal tobacco sales ban	https://www.sabcnews.com/sabcnews/sca-grants-fita-leave-to-appeal-tobacco-sales-ban/
384	2020-08-11	SABC News	Staff Reporter	FITA submits papers to the SCA as cigarette ban battle rages on	https://www.sabcnews.com/sabcnews/fita-submits-papers-to-the-sca-as-cigarette-ban-battle-rages-on/
385	2020-08-04	SABC News	Makgala Masiteng	Supreme Court of Appeal grants FITA leave to appeal tobacco judgment	https://www.sabcnews.com/sabcnews/supreme-court-of-appeal-grants-fita-leave-to-appeal-tobacco-judgment/
386	2020-07-31	SABC News	Katlego Nyoni	Farmers decry continued ban on tobacco products, ‘will result in massive job losses’	https://www.sabcnews.com/sabcnews/farmers-decry-continued-ban-on-tobacco-products-will-result-in-massive-job-losses/
387	2020-07-28	SABC News	Makgala Masiteng	FITA to petition SCA to hear appeal opposing tobacco ban	https://www.sabcnews.com/sabcnews/fita-to-petition-sca-to-hear-appeal-opposing-tobacco-ban/
388	2020-07-25	SABC News	Thabile Mbhele	Nationwide protest against tobacco ban	https://www.sabcnews.com/sabcnews/nationwide-protest-against-tobacco-ban/
389	2020-07-25	SABC News	Staff Reporter	Fita concerned about government’s transparency on tobacco ban	https://www.sabcnews.com/sabcnews/fita-concerned-about-governments-transparency-on-tobacco-ban/

390	2020-07-24	SABC News	Staff Reporter	FITA's bid to have tobacco sales ban lifted suffers another blow	https://www.sabcnews.com/sabcnews/fitas-bid-to-have-tobacco-sales-ban-lifted-suffers-another-blow/
391	2020-07-15	SABC News	Staff Reporter	FITA takes cigarette ban fight back to court	https://www.sabcnews.com/sabcnews/fita-takes-cigarette-ban-fight-back-to-court/
392	2020-07-09	SABC News	Staff Reporter	FITA granted permission to appeal tobacco sales ban	https://www.sabcnews.com/sabcnews/fita-granted-permission-to-appeal-tobacco-sales-ban/
393	2020-06-30	SABC News	Jabulani Baloi	Four suspected illicit cigarette smugglers arrested in Musina	https://www.sabcnews.com/sabcnews/four-suspected-illicit-cigarette-smugglers-arrested-in-musina/
394	2020-06-27	SABC News	Staff Reporter	FITA could appeal tobacco ban judgement: Tax Justice SA	https://www.sabcnews.com/sabcnews/fita-could-appeal-tobacco-ban-judgement-tax-justice-sa/
395	2020-06-26	SABC News	Staff Reporter	FITA mulls way forward after failed bid to overturn ban on tobacco product sales	https://www.sabcnews.com/sabcnews/fita-mulls-way-forward-after-failed-bid-to-overturn-ban-on-tobacco-product-sales/
396	2020-06-26	SABC News	Staff Reporter	FITA's tobacco sales case dismissed	https://www.sabcnews.com/sabcnews/fitas-tobacco-sales-case-dismissed/
397	2020-06-26	SABC News	Staff Reporter	FITA understands the court has to make a complicated judgment on lifting tobacco sales ban	https://www.sabcnews.com/sabcnews/fita-understands-the-court-has-to-make-a-complicated-judgment-on-lifting-tobacco-sales-ban/
398	2020-06-14	SABC News	Manelisi Dubase	Smokers protest in Cape Town over continued cigarette ban	https://www.sabcnews.com/sabcnews/smokers-protest-in-cape-town-over-continued-cigarette-ban/
399	2020-06-10	SABC News	Tshepiso Moche	Court hears tobacco products sales matter	https://www.sabcnews.com/sabcnews/court-hears-tobacco-products-sales-matter/
400	2020-06-09	SABC News	Staff Reporter	Legality of tobacco ban to be heard in court	https://www.sabcnews.com/sabcnews/legality-of-tobacco-ban-to-be-heard-in-court/

401	2020-06-06	SABC News	Mercedes Besent	President Ramaphosa gives clarity on decision to ban tobacco sales	https://www.sabcnews.com/sabcnews/president-ramaphosa-gives-clarity-on-decision-to-ban-tobacco/
402	2020-06-06	SABC News	Lubabalo Dada	Eastern Cape government warns of disastrous consequences if tobacco sales allowed	https://www.sabcnews.com/sabcnews/eastern-cape-government-warns-of-disastrous-consequences-if-tobacco-sales-allowed/
403	2020-06-03	SABC News	Staff Reporter	Cop nabbed in possession of a consignment of cigarettes	https://www.sabcnews.com/sabcnews/cop-nabbed-in-possession-of-a-consignment-of-cigarettes/
404	2020-05-31	SABC News	Staff Reporter	Government made mistake in the way it communicated cigarette ban: Ramaphosa	https://www.sabcnews.com/sabcnews/government-made-mistake-in-way-it-communicated-cigarette-ban-ramaphosa/
405	2020-05-29	SABC News	Staff Reporter	BATSA to file an urgent court application against tobacco sales ban	https://www.sabcnews.com/sabcnews/british-american-tobacco-to-file-an-urgent-court-application/
406	2020-05-28	SABC News	Staff Reporter	AfriForum gives Dlamini-Zuma ultimatum as battle over cigarette sale ban rages on	https://www.sabcnews.com/sabcnews/afriforum-gives-dlamini-zuma-ultimatum-as-battle-over-cigarette-sale-ban-rages-on/
407	2020-05-24	SABC News	Staff Reporter	Alcohol sale to be allowed under level 3 lockdown, tobacco sales remain banned	https://www.sabcnews.com/sabcnews/alcohol-sale-to-be-allowed-under-level-3-lockdown-tobacco-sales-remain-banned/
408	2020-05-23	SABC News	Thabile Mbhele	Ethiopian busted with thousands cartons of illicit cigarettes	https://www.sabcnews.com/sabcnews/ethiopian-busted-with-thousands-cartons-of-illicit-cigarettes/
409	2020-05-16	SABC News	Staff Reporter	7 arrested for illegal possession of counterfeit cigarettes, hair weaves	https://www.sabcnews.com/sabcnews/7-arrested-for-illegal-possession-of-counterfeit-cigarettes-hair-weaves/
410	2020-05-12	SABC News	Tshepiso Moche	Fita halts legal action over ban on sale of cigarettes	https://www.sabcnews.com/sabcnews/fita-halts-legal-action-over-ban-on-sale-of-cigarettes/

411	2020-05-11	SABC News	Sashin Naidoo	Smokers share their struggles and victories amid tobacco sale ban	https://www.sabcnews.com/sabcnews/smokers-share-their-struggles-and-victories-amid-tobacco-sale-ban/
412	2020-05-06	SABC News	Staff Reporter	Liquor association urges easing on alcohol ban	https://www.sabcnews.com/sabcnews/liquor-association-urges-easing-on-alcohol-ban/
413	2020-05-06	SABC News	Tshepiso Moche	BATSA aborts decision to pursue legal action cigarette sale ban	https://www.sabcnews.com/sabcnews/batsa-aborts-decision-to-pursue-legal-action-cigarette-sale-ban/
414	2020-04-30	SABC News	Busi Chimombe	Analyst calls for transparency on government's sale of cigarettes decision	https://www.sabcnews.com/sabcnews/analyst-calls-for-transparency-on-governments-sale-of-cigarettes-decision/
415	2020-04-30	SABC News	Sashin Naidoo	Smokers slam government decision to extend ban on cigarettes	https://www.sabcnews.com/sabcnews/smokers-slam-government-decision-to-extend-ban-on-cigarettes/
416	2020-04-29	SABC News	Staff Reporter	Sale of cigarettes will not be allowed under Level-4 lockdown : Dlamini-Zuma	https://www.sabcnews.com/sabcnews/sale-of-cigarettes-will-not-be-allowed-under-level-4-lockdown-dlamini-zuma/
417	2020-04-24	SABC News	Staff Reporter	NCAS warns of harms associated to cigarettes as ban on sales lifted	https://www.sabcnews.com/sabcnews/ncas-warns-of-harms-associated-to-cigarettes-as-ban-on-sales-lifted/
418	2020-04-17	SABC News	Staff Reporter	Ban on alcohol sales to remain during lockdown	https://www.sabcnews.com/sabcnews/ban-on-alcohol-sales-to-remain-during-lockdown/
419	2020-04-17	SABC News	Horisani Sithole	Police crack-down on illegal sale of alcohol and cigarettes	https://www.sabcnews.com/sabcnews/police-crack-down-on-illegal-sale-of-alcohol-and-cigarettes/
420	2020-04-06	SABC News	Wendy Mothata	FITA urges government to lift the cigarettes ban amid lockdown	https://www.sabcnews.com/sabcnews/fita-urges-government-to-lift-the-cigarettes-ban-amid-lockdown/
421	2020-04-01	SABC News	Horisani Sithole	South Africans addicted to alcohol, cigarettes negatively affected by lockdown	https://www.sabcnews.com/sabcnews/south-africans-addicted-to-alcohol-and-cigarettes-negatively-affected-by-lockdown/

422	2020-09-16	SABC News	Lerato Matlala	South Africa moves to Lockdown Level one	https://www.sabcnews.com/sabcnews/south-africa-moves-to-lockdown-level-one/
423	2020-08-12	SABC News	Busi Chimombe	DA calls for complete lifting of lockdown	https://www.sabcnews.com/sabcnews/da-calls-for-complete-lifting-of-lockdown-2/
424	2020-08-07	SABC News	Ismael Modiba	Pretoria High Court correct to dismiss FITA's application: Government	https://www.sabcnews.com/sabcnews/pretoria-high-court-was-correct-to-dismiss-fitas-application-government/
425	2020-08-06	SABC News	Staff Reporter	Government has until Friday to file its response to FITA's application	https://www.sabcnews.com/sabcnews/government-has-until-friday-to-file-its-response-to-fitas-application/
426	2020-06-26	The Citizen	Bernadette Wicks	Bad news for smokers: High court says cigarette ban was rational	https://citizen.co.za/news/covid-19/breaking-news-covid-19/2309965/bad-news-for-smokers-high-court-says-cigarette-ban-was-rational/
427	2020-06-03	The Citizen	Citizen reporter	Court gives govt till Friday 5pm to respond on tobacco ban	https://citizen.co.za/business/business-news/2296301/court-gives-govt-till-friday-5pm-to-respond-on-tobacco-ban/
428	2020-08-05	The Citizen	Gopolang Moloko	Batsa tobacco ban case against govt 'defining moment' for SA – Tax Justice SA	https://citizen.co.za/news/south-africa/courts/2335412/batsa-tobacco-ban-case-against-govt-defining-moment-for-sa-tax-justice-sa/
429	2020-10-29	The Citizen	Rorisang Kgosana	Extended tobacco ban contributed to low tax revenue as debt rises	https://citizen.co.za/business/business-news/2376655/extended-tobacco-ban-contributed-to-low-tax-revenue-as-debt-rises/
430	2020-05-04	The Citizen	Professor Francois Steyn and Harriet Klopper	Tobacco ban fuels illegal trade	https://citizen.co.za/business/business-news/2278209/tobacco-ban-fuels-illegal-trade/

431	2020-08-11	The Citizen	Bernadette Wicks	Tobacco ban is killing smokers	https://citizen.co.za/news/south-africa/courts/2339240/tobacco-ban-is-killing-smokers/
432	2020-05-29	The Citizen	Staff Reporter	British American Tobacco to take legal action over cigarette ban	https://citizen.co.za/business/2293273/british-american-tobacco-to-take-legal-action-over-cigarette-ban/
433	2020-08-18	The Citizen	Gopolang Moloko	IN PICS: As smokers, boozers flood outlets, Fita notes possible price hike	https://citizen.co.za/news/south-africa/society/2343996/in-pics-as-smokers-boozers-flood-outlets-fita-notes-possible-price-hike/
434	2020-05-28	The Citizen	Makhosandile Zulu	Malema says attacks on Dlamini-Zuma and claims of ties to Mazzotti are 'nonsensical'	https://citizen.co.za/news/covid-19/2292753/malema-says-attacks-on-dlamini-zuma-and-claims-of-ties-to-mazzotti-are-nonsensical/
435	2020-05-07	The Citizen	Staff Reporter	Government will not publish cigarette ban U-turn minutes due to 'classified' status	https://citizen.co.za/news/south-africa/government/2280543/government-will-not-publish-cigarette-ban-u-turn-minutes-due-to-classified-status/
436	2020-06-10	The Citizen	Bernadette Wicks	Fita argues against government's 'obsession' with cigarettes	https://citizen.co.za/news/south-africa/courts/2300064/watch-fita-argues-against-governments-obsession-with-cigarettes/
437	2020-06-02	The Citizen	Rorisang Kgosana	Smokers' march goes up in smoke	https://citizen.co.za/premium/2295565/smokers-march-goes-up-in-smoke/
438	2020-07-29	The Citizen	Staff Reporter	Smoking', 'lack of social distancing' at Mlangeni's funeral causes uproar on Twitter	https://citizen.co.za/news/south-africa/social-media/2331317/smoking-lack-of-social-distancing-at-mlangenis-funeral-causes-uproar-on-twitter/
439	2020-03-30	The Conversation	Benjamin T H Smart and Alex Broadbent	SA's Covid-19 lockdown: Smokes and outdoor exercise could ease the tension	https://theconversation.com/south-africas-covid-19-lockdown-cigarettes-and-outdoor-exercise-could-ease-the-tension-134931

440	2020-06-03	TimesLI VE	Ernest Mabuza	Ban on cigarettes will not make people quit smoking, Fita tells high court	https://www.timeslive.co.za/news/south-africa/2020-06-03-ban-on-cigarettes-will-not-make-people-quit-smoking-fita-tells-high-court/
441	2020-05-28	TimesLI VE	Shonisani Tshikalange	Cardiologist questions government ban aimed at smokers	https://www.timeslive.co.za/news/south-africa/2020-05-28-cardiologist-questions-government-ban-aimed-at-smokers/
